# Machine learning and molecular docking prediction of potential inhibitors against dengue virus

**DOI:** 10.3389/fchem.2024.1510029

**Published:** 2024-12-24

**Authors:** George Hanson, Joseph Adams, Daveson I. B. Kepgang, Luke S. Zondagh, Lewis Tem Bueh, Andy Asante, Soham A. Shirolkar, Maureen Kisaakye, Hem Bondarwad, Olaitan I. Awe

**Affiliations:** ^1^ Department of Parasitology, Noguchi Memorial Institute for Medical Research (NMIMR), College of Health Sciences (CHS), University of Ghana, Accra, Ghana; ^2^ Department of Biochemistry, Faculty of Sciences, University of Douala, Douala, Cameroon; ^3^ Pharmaceutical Chemistry, School of Pharmacy, University of Western Cape Town, Cape Town, South Africa; ^4^ Department of Computer Engineering, Faculty of Engineering and Technology, University of Buea, Buea, Cameroon; ^5^ Department of Immunology, Noguchi Memorial Institute for Medical Research (NMIMR), College of Health Sciences (CHS), University of Ghana, Accra, Ghana; ^6^ College of Engineering, University of South Florida, Florida, United States; ^7^ Department of Immunology and Molecular Biology, College of Health Sciences, Makerere University, Kampala, Uganda; ^8^ Department of Biotechnology and Bioinformatics, Deogiri College, Dr. Babasaheb Ambedkar Marathwada University, Sambhajinagar, India; ^9^ African Society for Bioinformatics and Computational Biology, Cape Town, South Africa

**Keywords:** molecular docking, drug discovery, machine learning, dengue virus, molecular dynamics simulation

## Abstract

**Introduction:**

Dengue Fever continues to pose a global threat due to the widespread distribution of its vector mosquitoes, *Aedes aegypti* and *Aedes albopictus*. While the WHO-approved vaccine, Dengvaxia, and antiviral treatments like Balapiravir and Celgosivir are available, challenges such as drug resistance, reduced efficacy, and high treatment costs persist. This study aims to identify novel potential inhibitors of the Dengue virus (DENV) using an integrative drug discovery approach encompassing machine learning and molecular docking techniques.

**Method:**

Utilizing a dataset of 21,250 bioactive compounds from PubChem (AID: 651640), alongside a total of 1,444 descriptors generated using PaDEL, we trained various models such as Support Vector Machine, Random Forest, k-nearest neighbors, Logistic Regression, and Gaussian Naïve Bayes. The top-performing model was used to predict active compounds, followed by molecular docking performed using AutoDock Vina. The detailed interactions, toxicity, stability, and conformational changes of selected compounds were assessed through protein-ligand interaction studies, molecular dynamics (MD) simulations, and binding free energy calculations.

**Results:**

We implemented a robust three-dataset splitting strategy, employing the Logistic Regression algorithm, which achieved an accuracy of 94%. The model successfully predicted 18 known DENV inhibitors, with 11 identified as active, paving the way for further exploration of 2683 new compounds from the ZINC and EANPDB databases. Subsequent molecular docking studies were performed on the NS2B/NS3 protease, an enzyme essential in viral replication. ZINC95485940, ZINC38628344, 2′,4′-dihydroxychalcone and ZINC14441502 demonstrated a high binding affinity of −8.1, −8.5, −8.6, and −8.0 kcal/mol, respectively, exhibiting stable interactions with His51, Ser135, Leu128, Pro132, Ser131, Tyr161, and Asp75 within the active site, which are critical residues involved in inhibition. Molecular dynamics simulations coupled with MMPBSA further elucidated the stability, making it a promising candidate for drug development.

**Conclusion:**

Overall, this integrative approach, combining machine learning, molecular docking, and dynamics simulations, highlights the strength and utility of computational tools in drug discovery. It suggests a promising pathway for the rapid identification and development of novel antiviral drugs against DENV. These *in silico* findings provide a strong foundation for future experimental validations and *in-vitro* studies aimed at fighting DENV.

## 1 Introduction

Dengue Virus (DENV) is a positive-sense ssRNA virus belonging to the family *Flaviviridae*, responsible for the most prevalent viral hemorrhagic fever transmitted by mosquitoes (Chao et al., 2018). The disease is transmitted to humans by the mosquitoes *Aedes aegypti* and *Aedes albopictus*, especially in hyperendemic regions in Southeast Asia and the Pacific experience the cocirculation of multiple serotypes of the virus (Caminade et al., 2012; Cucunawangsih and Lugito, 2017). There are four unique DENV serotypes (DENV1, DENV2, DENV3, and DENV4); historically, these four serotypes circulated in different geographic areas (Jamal et al., 2024; Murray et al., 2013). The prevalence rates have been deemed 390 million cases as of 2024, with 96 million being symptomatic (Anasir and Ramanathan, 2020; Rachmawati et al., 2024) and annual death recorded at around 25,000 (Yadouleton et al., 2024). The impact of Dengue fever is at its peak in North and South America, the Southeastern part of Asia, and the Western Pacific (Gebhard et al., 2019). Its symptoms usually are myalgia, hemorrhagic features, arthralgia, headache, rash, and retro-orbital discomfort (Chikkaveeraiah et al., 2024; Drago et al., 2021). In severe cases, it may also lead to Dengue Hemorrhagic Fever (DHF) and Dengue Shock Syndrome (DSS), which is an acute vascular permeability syndrome (Chikkaveeraiah et al., 2024). The probability of the disease transitioning into DHF and DSS is considerably higher for patients who have developed secondary DENV infections, around 10 to 100-fold (Martina et al., 2009).

The DENV genome is 11 kb long, comprising 10,723 nucleotides, and encodes large polyprotein precursors of approximately 3,391 amino acid residues (Gautam et al., 2024). After being cleaved by host and viral proteases, these DENV polyproteins form three structural proteins: C, prM, and E (where each stands for capsid, pre-membrane, and envelope, respectively) as well as seven non-structural proteins (NSPs): NS1, NS2A, NS2B, NS3, NS4A, NS4B, and NS5 (Dwivedi et al., 2017). The structural and non-structural proteins of the viral genome have all been identified as potential drug targets against Dengue infection (M. F. Lee et al., 2024). However, among these proteins, the envelope protein and the NSPs, NS3, and NS5 proteins have been identified as the proteins that play a vital role in viral replication (Chen et al., 2018; M. F. Lee et al., 2024). Due to mutations in specific proteins of the virus, emerging resistance to existing therapeutics has been reported (S. P. Lim, 2019) and thus calls for the urgent need to identify multiple vital drug targets that can effectively halt the replication of the virus in its host.

Significant efforts to contain the spread of Dengue fever can be seen in vaccine development, vector control mechanisms, and efforts to reduce viral load and preventive measures against severe forms of Dengue infection (M. F. Lee et al., 2024). Low et al. (2011) discovered that Narasin is a novel antiviral agent effective against all DENV serotypes with an IC50 of less than 1 μM (Gautam et al., 2024). Brefeldin is a promising antiviral compound with a 54.6–65.7 nM IC50 range for all DENV serotypes (Raekiansyah et al., 2017). There have been research efforts that aimed to model the evolution of viral pathogens like SARS-CoV-2 using genomic sequence data (Awe et al., 2023), HIV-1 evolution in sub-Saharan Africa (Obura et al., 2022) and Ebola Virus using comparative genomics (Oluwagbemi and Awe, 2018). Additionally, several experimental studies have evaluated the activity of repurposed drugs against DENV. Therefore, it is essential to continue exploring more elements and inhibitors to develop potent antivirals with high efficacy against DENV (Punekar et al., 2022). Despite decades of attempts to discover new drugs and vaccines, Dengvaxia is the sole vaccine accepted against DENV (marketed in several countries). Dengvaxia has been noted to be non-efficacious against certain dengue strains which dropped its efficacy rate to 61% (X.-N. Lim et al., 2019; Pintado Silva and Fernandez-Sesma, 2023; Thomas, 2023). TAK-003 and Butantan-DV are newly developed live-attenuated vaccines against DENV that have completed their phase III clinical trials, but the data regarding their efficacy against DENV3 and DEN4 is still insufficient (Biswal et al., 2020; Durbin, 2020; Kallas et al., 2020). As effective prophylactic and therapeutic measures against DENV are not present, the focus of patient management diverts to supportive therapy and controlling further transmission with drugs such as Chloroquine and Prednisolone (Lai et al., 2017).

Machine learning models like Support Vector Machine, Random Forest, Logistic Regression, and Naive Bayesian have been extensively applied in drug discovery, bioinformatics, and cheminformatics (Aniceto et al., 2023; Das et al., 2024; Niazi and Mariam, 2024). Advances in next-generation sequencing also enable the application of bioinformatics in diverse fields in the biomedical sciences and in applications like biomarker discovery (Chikwambi et al., 2023; Nyamari et al., 2023; El Abed et al., 2023; Ben et al., 2024; Alaya et al., 2024), co-infection biomarkers of parasites and viruses (Nzungize et al., 2022), analysis of RNA-seq, ChIP-seq data (Ather et al., 2018), genetics of complex diseases (Abolo et al., 2024) and in agriculture (Die et al., 2019; Omar et al., 2024), protein structure prediction (Pawar et al., 2024) and genomics applications in newborn screening (Wesonga and Awe, 2022). Various researchers used different ML techniques to study DENV, such as Gradient Boosting Machine (GBM), Random Forest (RF), and Support Vector Machine (SVM). Sanchez-Gendriz used an interesting technique (Sanchez-Gendriz et al., 2022) in which he developed a neural networking model with Long Short-Term Memory (LSTM) as the base for his studies in predicting future dengue cases in America. Another interesting study (Andersson et al., 2018) used a Convolution Neural Network to process street-level photos to predict DF and DHF rates in urban areas.

This study sought to build different machine learning models using the DENV2 CPE-Based HTS dataset from PubChem to distinguish between potential anti-dengue and non-anti-dengue compounds. The best-performing model based on the accuracy, specificity, Precision, and F1 score was used to predict active compounds solicited from the AfroDb (Ntie-kang et al., 2013), a catalog of ZINC15 database (Sterling and Irwin, 2015) and compounds present in the East African Natural Product Database (EANPDB) (Simoben et al., 2020). The predicted active compounds were further corroborated by employing molecular docking studies. The most promising drug candidates amongst the predicted compounds from our trained model were highlighted while also visualizing the intermolecular interactions between key residues in the active site and the compounds. The noxiousness of the compounds was estimated using SwissADME and DataWarrior. Molecular Dynamics (MD) simulations with Molecular Mechanics Poisson-Boltzmann Surface Area (MMPBSA) were utilized to evaluate predicted leads.

## 2 Methods

**FIGURE 1:**
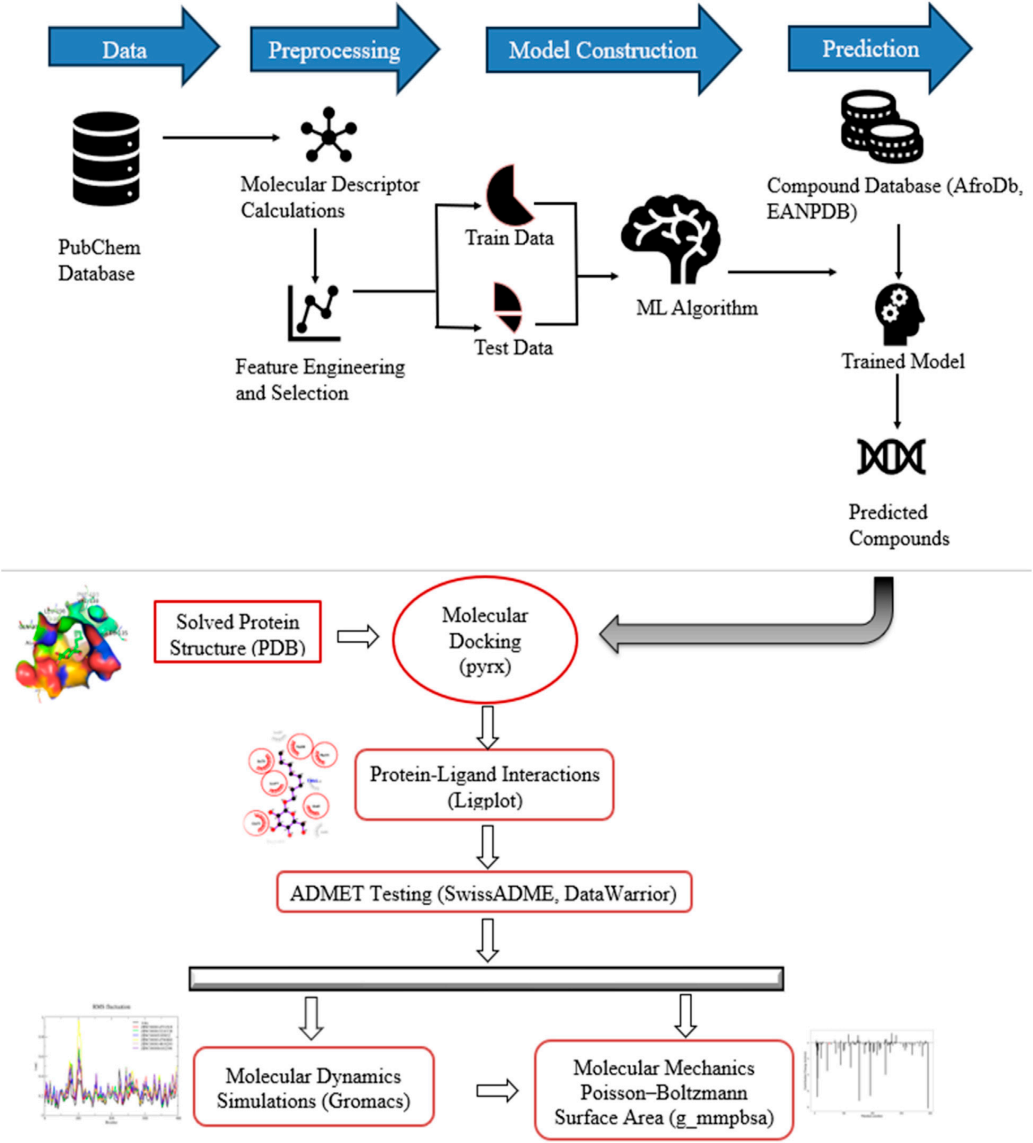
Graphical illustration of study workflow methods and instruments. The study developed five models on data from PubChem and used it for predicting new compounds. The predicted hits were screened through molecular docking, *in silico* pharmacological and toxicity tests, structural assessment using MD simulations, and estimation of binding free energies.

A visual representation of the methodology applied to this study is presented (Figure 1).

### 2.1 Dataset acquisition

This study proceeded in two phases as illustrated in the graphical depiction in Figure 1. First, a high throughput screen (HTS) measured in Cell-Based and Microorganism Combination System bioassay data of 343,305 compounds retrieved from PubChem was used for the study. The dataset with PubChem AID: 651640 was aimed to identify inhibitors of Dengue Virus by treating BHK-21 with various compounds before being infected with Dengue Virus serotype 2. The DENV2 strain New Guinea C, obtained from the American Type Culture Collection (ATCC) with catalog number VR-1584 was adapted (Che et al., 2009). Several viruses have been successfully studied using the cytopathic effect (CPE) assay to find new antiviral substances (Lin et al., 2023; McCormick et al., 2012).The expected outcome was that compounds increased ATP levels, indicating increased cell viability compared to the positive control (uninfected cells), and the neutral control was considered positive and otherwise negative. The dataset retrieved for this study comprised 5,946 active and 321,638 inactive compounds, a ratio of about 1:50 compounds. To compute molecular descriptors, the actives and inactives of the dataset were downloaded in the Structure Data Format (SDF).

### 2.2 Calculation of molecular descriptors and data preprocessing

Recent studies have demonstrated the utility of molecular descriptors in predicting compound activity, toxicity, and other properties from chemical structures (Comesana et al., 2022; Trinh et al., 2023). The active and inactive datasets were converted from SDF to Simplified Molecular-Input Line-Entry System (SMILES) formats for easier manipulation for machine learning. PaDEL-descriptor calculator (Yap, 2011) was used to compute 1440 molecular descriptors from the canonical SMILES of the compounds. Before descriptor calculations, compound standardization was performed, involving salt removal and nitro group normalization, to ensure uniformity and accuracy of the descriptor data (Viganò et al., 2024). The dataset used for training and testing the models comprised 4470 active compounds and 16780 inactive compounds, after reducing the ratio between the active and inactive compounds to 1:4 to enhance the computational efficiency.

The significant imbalance between active and inactive compounds was acknowledged, but oversampling techniques to balance the classes were not applied. A variance filter was employed to address the issue of dimensionality and enhance the relevance of features for predictive modeling (Velliangiri et al., 2019). Mean imputation was applied to handle any missing values within the dataset, ensuring a complete set of descriptors for each compound. Standardization of the dataset was done by removing the mean and scaling to unit variance using the Standard Scaler from the scikit-learn library. This transformation ensures that the features are centered around zero and have a standard deviation of one, which is crucial for many machine learning algorithms to perform optimally, as it ensures that the model is not biased towards features with larger numerical ranges. The was computed using the formula 1:

$$\text{Standard Scaler}\left(X\right)=\frac{X\;\left(i\right)-mean}{standard\;deviation\;}$$ (1)

Where i represent each value in the feature X.

### 2.3 Development of machine learning models

Five models were built from the training dataset using five machine learning algorithms and the model with superior performance in terms of classification metrics was chosen (Kee et al., 2023; Tougui et al., 2021). A 70%–30% split of the pre-processed data was used for training and testing the models, respectively. The classification models developed included Support Vector Machine (SVM), k-nearest neighbors (k-NN), Gaussian Naïve Bayes (GaussianNB) (Adams et al., 2022), Random Forest classifier (RF), and Logistic Regression (LR) (Khorshid et al., 2021). For the k-nearest neighbors (k-NN) model, k = 3 was used. The Gaussian Naïve Bayes model was implemented using default settings from the scikit-learn library (Pedregosa et al., 2011). The SVM model was optimized with the probability parameter set to True (Sandhu et al., 2022). The Random Forest model was built with a maximum depth of 8 and 100 estimators (J. Adams et al., 2022). Finally, the Logistic Regression model was constructed with a maximum iteration parameter of 1000.

### 2.4 Model validation

Prior to comparison with other optimized classifiers, each classifier underwent optimization to determine the optimal hyperparameters that yielded the maximum accuracy. The optimized models were evaluated using 10-fold cross-validation. This method splits the training data into kkk groups, trains the model on k-1k-1k-1 folds, and tests the model on the remaining fold to yield a trustworthy estimate of the model’s performance on unseen data (Jung and Hu, 2015; Vabalas et al., 2019). It computes performance metrics such as accuracy, accuracy, precision, recall, and F1 score based on the confusion matrix, which includes true positives (TP), true negatives (TN), false positives (FP), and false negatives (FN) (Tharwat, 2021). These metrics (as shown in Equations 2–6) were used to compare and select the best-performing model for predicting Dengue Virus inhibitors (Adams et al., 2022; Orozco-arias et al., 2020)

$$\text{Accuracy}=\frac{TP+TN}{TP+FP+TN\;FN}$$ (2)

$$\text{Precision}=\frac{TP}{TP+FP}$$ (3)

$$\text{Recall}=\frac{TP}{TP+FN}$$ (4)

$$F1=2\;x\;\frac{Precision\;*\;Recall\;}{Precision+Recall}$$ (5)

$$\text{Specificity}=\frac{TN}{TN+FP}$$ (6)

### 2.5 Prediction of compounds

The best-performing model was used to make predictions against 812 compounds from Afrodb, and 1871 EANPDB compounds. Prior to compound predictions, the model’s predictive power was validated on known Dengue Virus inhibitors post-cross-validation. The molecular descriptors of these inhibitors were calculated and preprocessed similarly to the training and test data. This was done to reinforce the credibility of the cross-validation results for the best-performing model based on the metrics. The number of correct predictions made by the model on the submitted inhibitors defines the accuracy of the classification or prediction. A total of 18 known inhibitors curated from literature were submitted to the LR model as a result of their activity against the Dengue Virus shown in different studies.

### 2.6 Preparation of target protein and ligand libraries

The Crystal structure of the Dengue 2 Virus nonstructural protein NS2B/NS3 was obtained from the RCSB Protein Data Bank (http://www.rcsb.org/pdb) with PDB ID: 2FOM. Before selecting 2FOM, different suitable structures such as 4M9M, 4M9T, and 4M9I were retrieved, however, 2FOM was chosen based on its low resolution, low R values, and the number of missing residues. The selected protein structure was superimposed with the other suitable structure using PyMOL to measure their root mean square deviation (RMSD). The structure was cleaned using PyMOL (Yuan et al., 2017) to devoid the protein of ions, water molecules, and other structures like ligands before minimization was carried out by employing Groningen Machine for Chemical Simulations (GROMACS) (Abraham et al., 2015). The steepest descent minimization algorithm with a maximum number of 50,000 steps and a minimization step size of 0.01 was used to minimize the protein structure. The three-dimensional structures (.sdf) of the natural compounds predicted by the machine learning model were obtained for the molecular docking stage. Natural products were chosen for this investigation because of their structural and chemical variety, and the therapeutic effects of phytochemicals found in plants.

### 2.7 Molecular docking and mechanism of binding characterization

The molecular docking procedure for the predicted compounds was carried out using AutoDock Vina (Trott and Olson, 2010). All compounds were energy minimized in 200 steps using the Universal force field (UFF) before being translated to the Protein Data Bank partial charge and atom type (.pdbqt) format using Open Babel software (O’Boyle et al., 2011). Visualization of the resultant energy minimized protein structure and the removal of surrounding water molecules before the virtual screening was done in PyMOL v1.5.0.4. The prepared structure was then saved using PyMOL before applying the “make macromolecule” option in PyRx to prepare for the docking of selected hits. The library was screened against the NS2B/NS3 protease using a grid box dimension of center_x = −5.179Å center_y = −9.575Å center_z = 13.756Å size_x = 18.302Å size_y = 19.821Å size_z = 23.788Å.

All hit compounds that contributed binding affinities of at least −8.0 kcal/mol were considered. The output of AutoDock Vina is ranked in a decreasing order of binding affinity using a negative function; a more negative binding affinity is preferred. The best mode for each compound was applied using the root mean squared deviation. The result was then examined with PyMOL to find the optimal-docked ligands. LigPlot + (v1.4.5) was used to analyze the interactions between key residues in the active site of the protein and the docked compounds (Laskowski and Swindells, 2011). The protein-ligand complexes generated in PyMOL were used as input for LigPlot. The resulting output provides a 2D depiction of intermolecular interactions, including hydrophobic interactions and hydrogen bonds.

### 2.8 ADMET screening of selected compounds

A pharmacokinetics profile, comprising an assessment of absorption, distribution, metabolism, and excretion (ADME), was applied to a subset of compounds using SwissADME (Daina et al., 2017). Along with Veber’s rule, the ADMET testing also measured five properties: total average molecular weight in g/mol, the number of hydrogen bond donors, hydrogen bond acceptors, rotatable bonds, and partition coefficient—collectively referred to as Lipinski’s rule of five (Devadasu et al., 2018; Jia et al., 2020). Using OSIRIS DataWarrior v06.02.05 (Sander et al., 2015), the toxicity characteristics, including mutagenicity and tumorigenicity, were predicted.

### 2.9 Molecular dynamics (MD) simulations

A 100 ns MD simulation was run on each protein-ligand complex and the unbound protein using GROMACS-2020.5 on a Dell EMC high-performance computing cluster at the WACCBIP, University of Ghana, Accra. The CHARMM36 all-atom force field produced the protein and ligand topology (July 2022). Utilizing a cubic box for all simulations, the systems were each solvated with water molecules, neutralized with ions, and energy-minimized to optimize the system. To equilibrate each system, NVT, and NPT ensemble were applied for 100 ps a piece before the production run. The parameters for the production run included 50,000,000 steps which translates into 100 ns and a step size of 0.002 (2 fs). Xmgrace (Turner, 2005) was used to visualize and analyze the root mean square deviation (RMSD), root mean square fluctuation (RMSF), and radius of gyration (Rg) obtained from the MD simulations.

### 2.10 Calculations of MMPBSA parameters

The binding free energies of the protein-ligand complexes and the individual energy contributions of the residues were calculated using the Molecular Mechanics Poisson-Boltzmann Surface Area (MMPBSA) method (Kumari et al., 2014). This is a corroboration technique used to verify the limitations of the existing scoring function utilizing the MD simulation output files (Wang C. et al., 2018). R programming software was utilized to plot the graphs from the MMPBSA computations.

## 3 Results

### 3.1 Data acquisition and processing

**FIGURE 2:**
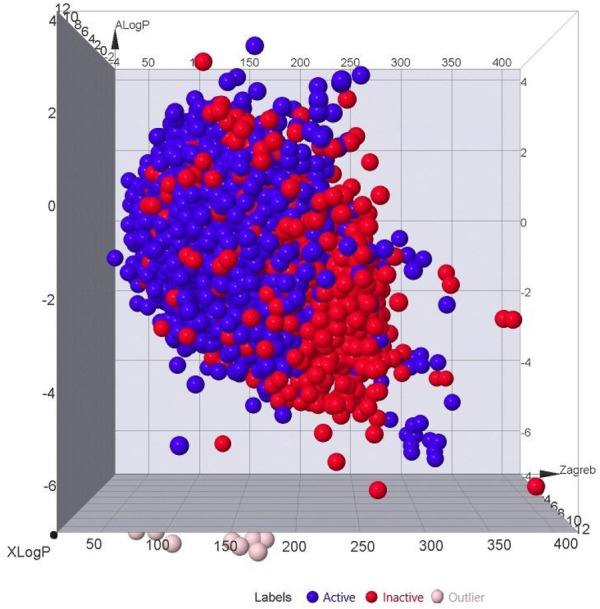
3D plot showing the correlation between the active and inactive compounds based on ALogP, XLogP, and Zagreb. [Labels: Blue = Actives, Red = Inactives, Pink = Outliers].

The bioactive dataset obtained from PubChem was an imbalanced set, with approximately one-third of its constituents being active compounds. As displayed in Figure 2, inactive compounds dominated the dataset. Using PaDEL, 1,444 molecular descriptors were generated, which provide a mathematical representation of the compounds for QSAR modeling by converting chemical information about the compounds into numerical values. The dataset underwent a three-dataset splitting strategy to be divided into training, validation, and test sets. The dataset of 21,250 compounds was split into 14,875 training data, 3,187 test data, and 3,188 externally held data. The first set is for training the algorithm, the validation set tunes hyperparameters, and the test set is used to test model performance and predicting ability. The application of a variance filter reduced the number of descriptors from 1,444 to 684, using a variance threshold of 0.1 to filter out descriptors with minimal variance. This step ensured that only the most informative features were retained for subsequent modeling, as low-variance features are often less useful in distinguishing between classes (Velliangiri et al., 2019).

### 3.2 Model development and evaluation

Five machine learning models were developed to predict Dengue Virus inhibitors: k-Nearest Neighbors (k-NN), Gaussian Naïve Bayes (NB), Support Vector Machine (SVM), Random Forest (RF), and Logistic Regression (LR). Each model was evaluated using statistical metrics such as accuracy, precision, recall, and F1 score, as displayed in Table 1 with the confusion matrix shown in Supplementary Table S1.

**TABLE 1:** Comparison of model performance on withheld data sets; showing results for accuracy, precision, recall, and F1 scores.

Model	Accuracy	Precision	Recall	F1 score
LR	0.94	0.91	0.76	0.83
SVM	0.93	0.94	0.71	0.81
KNN	0.92	0.89	0.68	0.77
RF	0.91	0.94	0.6	0.73
NB	0.81	0.55	0.47	0.51

Among the models, Logistic Regression demonstrated the highest performance across most metrics, including an accuracy of 94%, a precision of 91%, and an F1 score of 0.83. This superior performance made Logistic Regression the most suitable model for predicting Dengue Virus inhibitors in this study. The SVM model followed closely, with an accuracy of 93%, a precision of 94%, and an F1 score of 0.81. Although the k-NN and Random Forest models performed well, they lagged behind the top two models in recall and F1 scores, indicating that they were less effective in identifying all active compounds.

Gaussian Naïve Bayes was the poorest-performing model, with an accuracy of 81% and an F1 score of 0.51. This model’s low precision (0.55) and recall (0.47) indicate that it struggled to balance identifying true positives and minimizing false positives, particularly in the imbalanced dataset where inactive compounds were predominant. Overall, Logistic Regression emerged as the most reliable model for predicting potential inhibitors due to its robust performance across the different metrics.

### 3.3 Prediction of known inhibitors and new compounds

The study further validated the performance of the developed models by testing 18 known Dengue Virus inhibitors curated from literature. The prediction made by the Logistic Regression model for these compounds is listed in Table 2. The Logistic Regression model correctly predicted 11 of the 18 inhibitors as active, outperforming other models, including SVM and Random Forest.

**TABLE 2:** Classification and mechanisms of action of known DENV inhibitors identified by the Logistic Regression model.

Number	Inhibitors	Prediction*	Mechanism of action	References
1	Pentoxifylline	1	Immune modulation	Salgado et al. (2012)
2	4-hydroxyphenyl retinamide	0	Inhibits viral replication	Carocci et al. (2015), Fraser et al. (2014)
3	Prochlorperazine	1	Inhibits viral binding and viral entry	Simanjuntak et al. (2015)
4	Balapiravir	1	Inhibits viral replication	Nguyen et al. (2013)
5	Bortezomib	1	Inhibits viral replication	Ci et al. (2023)
6	Leflunomide	1	Immunosuppressive effects	(W.-L. Wu et al., 2011)
7	SKI-417616	1	Inhibition of D4R suppressed DENV infection	Smith et al. (2014)
8	Celgosivir	1	Inhibits viral replication	Tian et al. (2018)
9	UV-4B	1	Inhibits viral replication	Franco et al. (2021)
10	2-C-methylcytidine	0	Inhibits viral replication	(J.-C. Lee et al., 2015)
11	Ketotifen	1	Vascular leakage	Lai et al. (2017)
12	Chloroquine	1	Inhibits viral replication	Lai et al. (2017)
13	Dasatinib	0	RNA replication inhibition	de Wispelaere et al. (2013)
14	Lovastatin	0	Inhibits viral replication	Whitehorn et al. (2016)
15	ST-148	0	Inhibits viral replication	Byrd et al. (2013)
16	Dexamethasone	0	Inhibits viral replication	Kularatne et al. (2009)
17	Prednisolone	1	Inhibits viral replication	Lai et al. (2017)
18	Ivermectin	0	Helicase inhibition	Xu et al. (2018)

*[0 = inactive; 1 = active].

Several inhibitors, such as Pentoxifylline, Prochlorperazine, and Balapiravir, were correctly classified as active by Logistic Regression, in line with their established mechanisms of action against the Dengue Virus. Notably, inhibitors like Celgosivir and Bortezomib, which inhibit viral replication, were also predicted accurately. This validation process of known inhibitors provided confidence in the model’s predictive capability, suggesting it could effectively generalize to novel compounds with similar mechanisms of action.

Following this validation, the Logistic Regression model was applied to predict 2,683 new compounds, including 812 from the ZINC database and 1,871 from the EANPDB database. Out of these, 933 compounds were predicted to be active, representing a promising pool of potential Dengue Virus inhibitors for further experimental validation.

These predictions highlight the utility of the developed QSAR models, particularly the Logistic Regression model, in identifying novel drug candidates for Dengue Virus inhibition. The robust performance on known inhibitors and newly predicted compounds highlights its potential as a valuable tool in future drug discovery efforts targeting the Dengue Virus. The study’s ability to handle imbalanced data effectively and generate accurate predictions underscores the importance of appropriate descriptor selection and data preprocessing in QSAR modeling.

### 3.4 Target selection and molecular docking of predicted compounds (PDB ID 2FOM)

**FIGURE 3:**
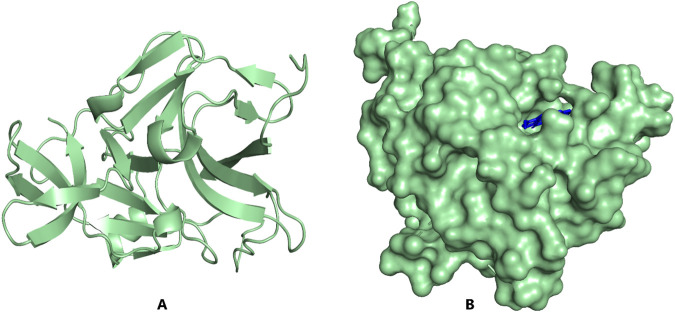
PyMOL visualization of NS2B/NS3 protease structure **(A)** Light-green cartoon structure representation; **(B)**. Light-green surface representation of the protein with ZINC000095486052 (blue) docked in the active site.

In this study, the NS2B/NS3 protease was preferentially selected amongst the seven nonstructural proteins of the Dengue Virus as the target structure to corroborate the prediction by the logistic regression model. The NS2B/NS3 protease is an essential enzyme for viral replication and assembly, making it a principal antiviral target for developing therapeutics against the virus (Erbel et al., 2006; Norshidah et al., 2023). There are two potential locations for inhibiting DENV protease: the active site and the blocking attachment of protease (NS3) to its protein cofactor (NS2B). The active site on the NS3 which is the prime target is made up of a conserved catalytic triad like His51-Asp75-Ser135 (Noble et al., 2012; Zamri et al., 2019). A search via the Protein Data Bank repository for a solved structure of the NS2B/NS3 for the Dengue Virus serotype showed IDs such as 4M9T, 2FOM, 4M9M, and 4M9I with resolutions 1.74, 1.50, 1.53, and 2.40 Å respectively and R-value work of 0.215, 0.176, 0.203, and 0.215 respectively. The 2FOM, solved using x-ray diffraction, was selected for this study since it had the lowest resolution and R-value, both of which are a measure of the quality of the structure (Wlodawer et al., 2008). Additionally, the RMSD values after superimposing the selected protein structure to 4M9T, 4M9M, and 4M9I were 0.358, 0.151, and 0.276 Å, respectively, underlying their close structural similarity with the 2FOM. The three-dimensional structure of the 2FOM with a ligand docked in the active site is shown Figure 3. The active site on the NS3 used in this study, besides consisting of the catalytic triad which is pivotal in inhibiting its activity, also consists of residues such as Leu128, Pro132, Ser131, and Tyr161 (Norshidah et al., 2023; Purohit et al., 2022).

**FIGURE 4:**
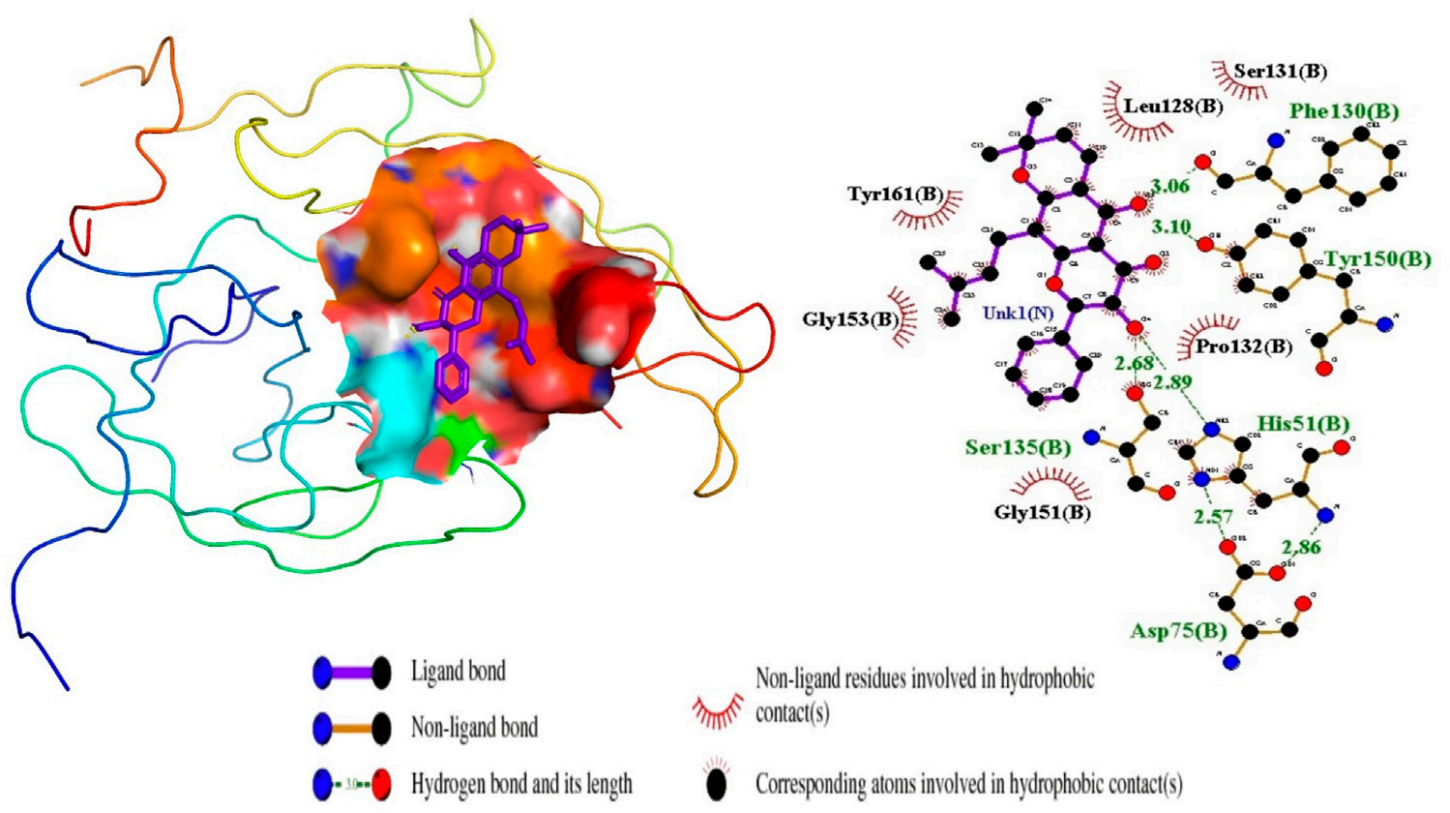
Ligand ZINC38628344 docked in NS2B/NS3 binding pocket; 3D pose and 2D protein-ligand interaction diagram generated using PyMOL and LigPlot, respectively.

A total of 853 compounds from the Logistic Regression prediction were docked into the active site of the target using Autodock. Anhydrophlegmacin showed the highest binding affinity of −9.2 kcal/mol towards the protease among all docked ligands. The docked compounds demonstrated binding affinities between −9.2 and −3.6 kcal/mol. This reinforces the prediction ability of the Logistic Regression model. Applying a threshold of −8.0 kcal, 59 ligands with affinities of −8.0 kcal/mol or better were considered for downstream analysis. A higher cutoff of −7.0 kcal/mol, taken as the standard threshold for a compound to be considered active against a particular target, was employed (Kwofie et al., 2019a). The higher the binding affinity, the stronger the bond between the ligands and the target protein. The protein in the complex with the ligands was visually inspected using PyMOL (Figure 4) to select the best-docked possess. In addition, inhibitors such as Leflunomide and Prednisolone were also incorporated into the docking to act as a control. They demonstrated affinities of −7.1 and −7.0 kcal/mol respectively. Table 3 shows the binding affinities of the top 20 of the selected compounds and the inhibitors.

**TABLE 3:** Protein-ligand interactions of top 20 hits with NS2B/NS3 post-docking, including interactions of two known inhibitors.

Compounds names	Binding affinity (kcal/mol)	Hydrogen bonding with bond length (Å)	Hydrophobic contacts
anhydrophlegmacin	−9.2	Asn152 (2.76), Gly153 (2.88), Ser135 (3.06), Gly151 (2.86)	Val72, Asp75, His51, Pro132, Tyr150, Leu128
anhydrophlegmacin-9,10-quinones_B2	−9.2	Val72 (2.96), Asp75 (2.57), His51 (2.86), Lys73 (2.94)	Leu128, Pro132, Gly151, Gly153, Tyr161, Trp50
ZINC000035941652	−9.1	Leu149 (3.06)	Trp83, Asn152, Ala164, Ile165, Lys73, Asn167, Thr120, Ile123, Ala166, Lys74, Gly148, Leu76
chryslandicin	−9	Val72 (2.74)	Gly153, Trp50, His51, Tyr161, Leu128, Pro132, Gly151, Asn152, Asp75
ZINC000085594516	−8.8	Ser135 (3.09)	Leu128, Tyr150, Pro132, Phe130, Gly151, His51, Asn152, G1y153, Asp75
6a,12a-dehydromillettone	−8.7	None	His151, Asp75, Gly151, Gly153, Tyr150, Phe130, Pro132, Leu128
ZINC000028462577	−8.6	Ser135 (2.67), Val72 (2.94)	Trp50, Gly151, Leu128, Phe130, His51, Gly153, Pro132, Tyr150
anhydrophlegmacin-9′,10′-quinone	−8.6	Asn152 (2.88), Gly153 (2.84), Ser135 (2.94)	Asp75, Val154, Val72, Trp50, His51, Pro132, Leu128, Gly151
2′,4′-dihydroxychalcone-(4-O-5‴)-4″,2‴,4‴-trihydroxychalcone	−8.6	Leu149 (2.99), Thr120 (3.26)	Val154, Lys73, Val72, Asn152, His51, Asp75, Gly148, Leu76, Gly153. Trp83, Lys74, Ile165, Ala166, Ala164, Asn167, Ile123
ZINC000095485910	−8.6	Phe130 (2.71)	Ser135, Gly151, Leu128, His51, Asp75, Gly153, Pro132, Tyr150
ZINC000095485955	−8.6	Trp83 (2.84), Leu149 (3.20), Asn152 (2.80)	Gly87, Val146, Met149, Leu76, Ala164, Asn167, Ile165, Ala166, Gly148, Leu85, Val147
ZINC000095486025	−8.5	Leu128 (3.34) Gly153 (2.87)	Val72, His51, Asp75, Ser135, Gly151, Phe130, Pro132, Tyr150, Tyr161, Val54, Lys73, Asn152
ZINC000038628344	−8.5	His51 (2.89), Ser135 (2.68), Asp75 (2.57), Phe130 (3.06), Tyr150 (3.10)	Pro132, Ser131, Leu128, Tyr161, Gly153, Gly151
ZINC000095486053	−8.4	Gly151 (2.99)	His51, Pro132, Tyr150, Ser135, Phe130, Leu128
phaseollidin	−8.4	Gly87 (2.83), Val146 (2.98)	Leu85, Trp83, Gly148, Leu149, Ala164, Leu76, Asn167. Asn152, Lys74, Ile165, Trp89, Ala166, Glu88, Glu86, Val147
6-oxoisoiguesterin	−8.4	Tyr150 (2.80, Phe130 (3.16, 2.83)	Ser131, Leu128, Gly151, Gly153, His51, Pro132
ZINC000095486052	−8.4	Asn152 (3.20), Gly153 (3.14)	Pro132, Tyr150, Leu128, Tyr161, Gly151, His151, Asp75
ZINC000014444870	−8.4	Asn152 (3.01), Leu149 (3.19)	Leu85, Val147, Gly87, Val146, Asn167, Ile165, Val54, Ala164, Ile123, Lys74, Gly148, Leu76, Trp83
Leflunomide	−7.1	None	Asn152, Val54, Ala64, Asn167, Leu76, Lys74, Ile123, Ala166
Prednisolone	−7.0	Gly151 (2.90, 2.71), Asp75 (2.95), His51 (3.21), Gly153 (2.93, 3.16)	Leu128, Phe130, Asn152, Ser135, Pro132

### 3.5 Mechanism of binding characterization of selected compounds

In continuation of the structure-based molecular docking to further confirm the binding affinities of the predicted compounds, the interactions of the compounds within the predicted binding pocket were determined. The biomolecular interactions between the NS2B/NS3 protease and the compounds were generated using LigPlot. Studies into these interactions are crucial in determining promising lead compounds. Identification of crucial residues in the corresponding targets’ active sites was made possible by characterizing the binding interactions. To find the suitable compound that inhibits the activities of the NS2B/NS3 protease, hydrogen and hydrophobic interactions between the shortlisted compounds and the residues in the active site were elucidated.

**FIGURE 5:**
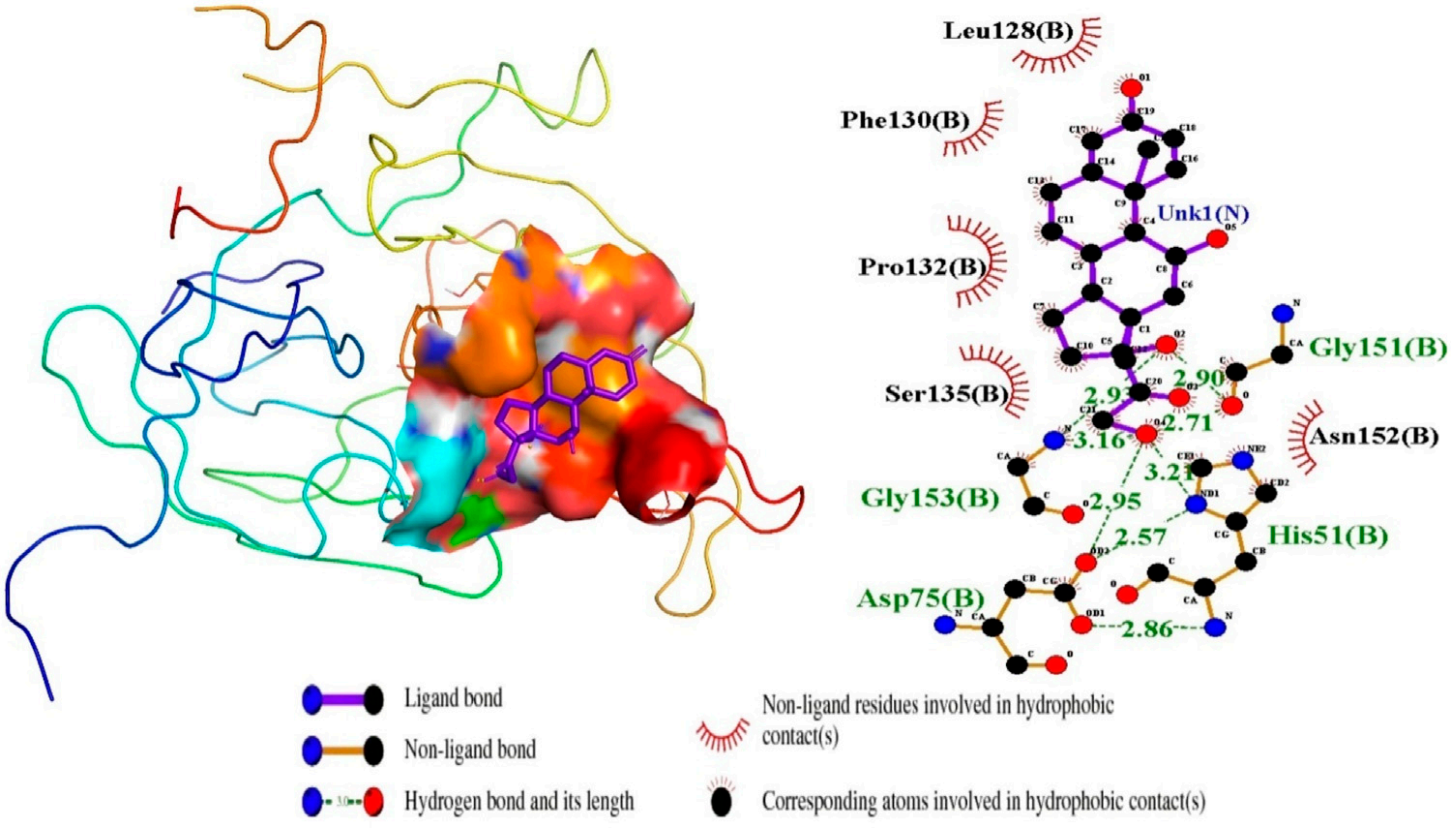
Inhibitor Prednisolone docked in the NS2B/NS3 binding pocket, showing the protein-ligand interactions visualized in LigPlot and the 3D pose in PyMOL.

For the interactions of the protease, the ligands docked to the active site were observed to interact with the proposed residues such as His51, Ser135, Leu128, Pro132, Ser131, Tyr161, and Asp75 as shown in Table 3 and Supplementary Table S2. Anhydrophlegmacin and anhydrophlegmacin-9,10-quinones_B2 which had the highest binding affinities interacted with similar residues such as His51, Asp75, Gly151, Leu128, Pro132, and Gly153. They interacted with conserved catalytic triad residues Asp75, Ser135, and His51 through hydrogen bonding with bond lengths of 2.57, 3.06, and 2.86 Å respectively. ZINC38628344, which had an affinity of −8.5 kcal/mol with the NS2B/NS3 protease formed hydrogen bond interaction with His51 (2.89 Å), Ser135 (2.68 Å), Asp75 (2.57 Å), Phe130 (3.06 Å), Tyr150 (3.10 Å) and hydrophobic interactions with residues Pro132, Ser131, Leu128, Tyr161, Gly153, Gly151 (Figure 4). The inhibitor Prednisolone interacted via hydrogen bonding with Gly151 (2.90 Å, 2.71 Å), Asp75 (2.95 Å), His51 (3.21 Å), and Gly153 (2.93 Å, 3.16 Å) as shown in Figure 5. In addition, ZINC14441502 formed hydrogen interactions with Gly151 and Ser135 with bond length 2.86 and 2.99 Å respectively; and hydrophobic bonding with Leu128, Gly153, Asn152, Val72, Asp75, His151 and Phe130 (Supplementary Table S2). 39 out of 56 hits docked firmly and interacted with critical residues in the active site, and these were selected for downstream analysis.

### 3.6 ADMET screening of selected compounds

Pharmacokinetics controls how medications are absorbed by the body and eventually eliminated (Wang G. Y. et al., 2018). Analyses were conducted on pharmacokinetic features, including gastrointestinal (GI) absorption. Drugs taken orally can enter the bloodstream through a process known as gastrointestinal absorption (GI) (Suenderhauf et al., 2012). “High” compound absorption occurs in the GI tract. To select druglike compounds, Veber’s criteria were also applied, and the selected hits that do not conform to Lipinski’s rule of five (RO5) were eliminated (Ogbodo et al., 2023). Out of the 39 hits, 20 were in violation of the rule (see Supplementary Table S3). Twelve hits also broke one of the RO5s. The remaining 7 hits: 5,7′-physcion-fallacinol, ZINC000095485956, ZINC000085594516, amentoflavone, ZINC000095486111, voucapane-18,19-di-(4-methyl)-benzenesulphonate, ZINC000095485927 showed the least drug-likeness of two RO5 violations (Table 4). Veberr’s rule, with TPSA ≤140 and rotatable bonds ≤10, was used as the main determinant (Veber et al., 2002). 26 out of the selected hits demonstrated 0 violations with the remaining showing only one violation of the rule. The solubility and pharmacological profiles such as GI absorption were also elucidated. Only ZINC000095485927 was predicted to be insoluble (Supplementary Table S3). Four of the 14 hits had a moderate solubility prediction and four had a soluble prediction. However, 19 of the selected hits were predicted to be poorly soluble (Supplementary Table S3). Compounds are considered to have met the GI absorption criteria if it is denoted as ‘High’ suggesting a high propensity of absorption into the intestinal tract for orally administered drugs. 21 and 18 of the selected hits were estimated to be high and low respectively. The mutagenicity and tumorigenicity levels of the hits were also predicted using DataWarrior (Table 4). From the results obtained, 26 out of the 39 hits tested were neither mutagenic nor tumorigenic.

**TABLE 4:** Prediction of ADME and toxicity profiles of top 15 selected hits.

Ligands	ESOL solubility class	GI absorption	RO5 violation	Veber’s rule violation	Mutagenicity	Tumorigenicity
ZINC000004095704	Soluble	Low	1	1	None	None
ZINC000095485958	Soluble	Low	1	1	None	None
ZINC000095485940	Soluble	High	0	0	None	None
ZINC000095485986	Soluble	Low	0	1	None	None
dihydrolanneaflavonol	Moderately soluble	High	0	0	None	None
lettowianthine	Moderately soluble	High	0	0	High	High
millettosine	Moderately soluble	High	0	0	None	None
ZINC000095486053	Moderately soluble	High	0	0	None	None
ZINC000031168265	Soluble	High	0	0	None	None
ZINC000095485910	Moderately soluble	High	0	0	High	High
ZINC000014780240	Moderately soluble	High	0	0	High	None
ZINC000085594516	Poorly soluble	Low	2	1	None	None
5,7′-physcion-fallacinol	Poorly soluble	Low	2	1	Low	None
ZINC000014441502	Moderately soluble	High	0	0	None	None
chryslandicin	Poorly soluble	Low	1	1	None	High

### 3.7 Molecular dynamics simulations

Molecular dynamics simulations were carried out using GROMACS 2020.5 to further elucidate the stability of the predicted lead compounds within the active site of the NS2B/NS3 protein (Mazumder et al., 2017). Understanding the binding mechanisms of the various molecules in the active site is crucial for the design of efficacious drugs. To analyze the dynamic behavior of the unbound proteins and complexes, the root mean square deviation, the radius of gyration, and the root mean square fluctuation were plotted with the use of Xmgrace (Agyapong et al., 2021; Kwofie et al., 2019b; Musyoka et al., 2016). All simulations were carried out for 100 ns.

#### 3.7.1 Root mean square deviation (RMSD)

**FIGURE 6:**
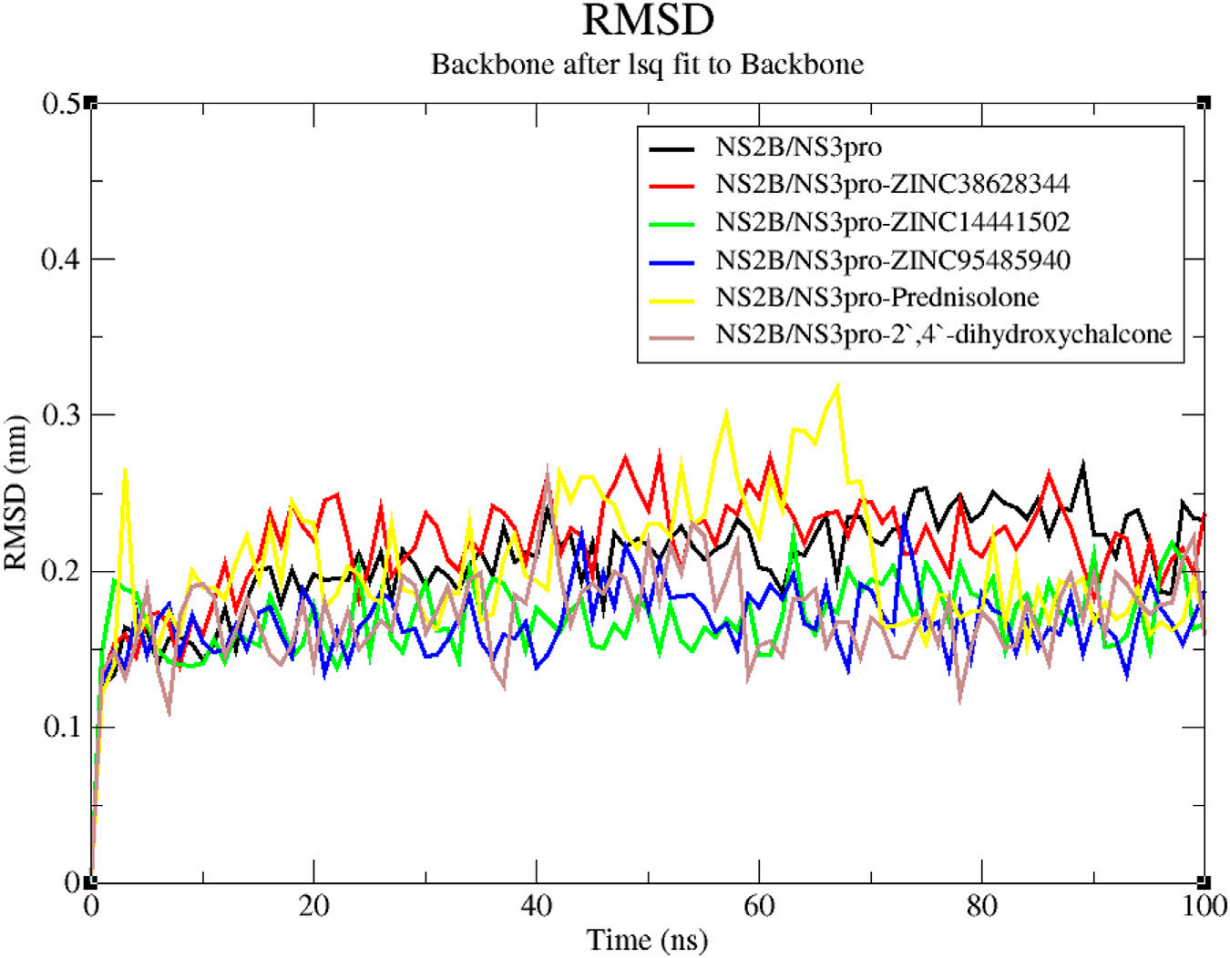
RMSD *versus* time graph of unbound protein and NS2B/NS3pro-ligand complexes generated over a 100 ns MD run.

A reliable indicator of a protein’s stability is the RMSD, which assesses the stability of the complex from the original protein backbone atomic coordinates (Adinortey et al., 2022; Kwofie et al., 2022). From the RMSD plot, the unbound protein and the four lead compounds experienced stability throughout the 100 ns run except for the inhibitor Prednisolone which was observed to be unstable till 70 ns of the run. The unbound protein was observed to have the least fluctuations. The RMSD plot for the NS2B/NS3pro-Prednisolone complex rose sharply from 0 to 0.26 nm after which it remained relatively unstable with large fluctuations until 70 ns where it demonstrated some stability (Figure 6). The RMSD of the NS2B/NS3pro-ZINC38628344 complex increased to 0.25 nm and stabilized, averaging 0.22 nm until the end. The complexes NS2B/NS3pro-ZINC95485940, NS2B/NS3pro-ZINC14441502, and NS2B/NS3pro-2′,4′-dihydroxychalcone showed similar fluctuations with the RMSD averaging around 0.17 nm (Figure 6).

#### 3.7.2 Radius of gyration for 100 ns MD simulations

**FIGURE 7:**
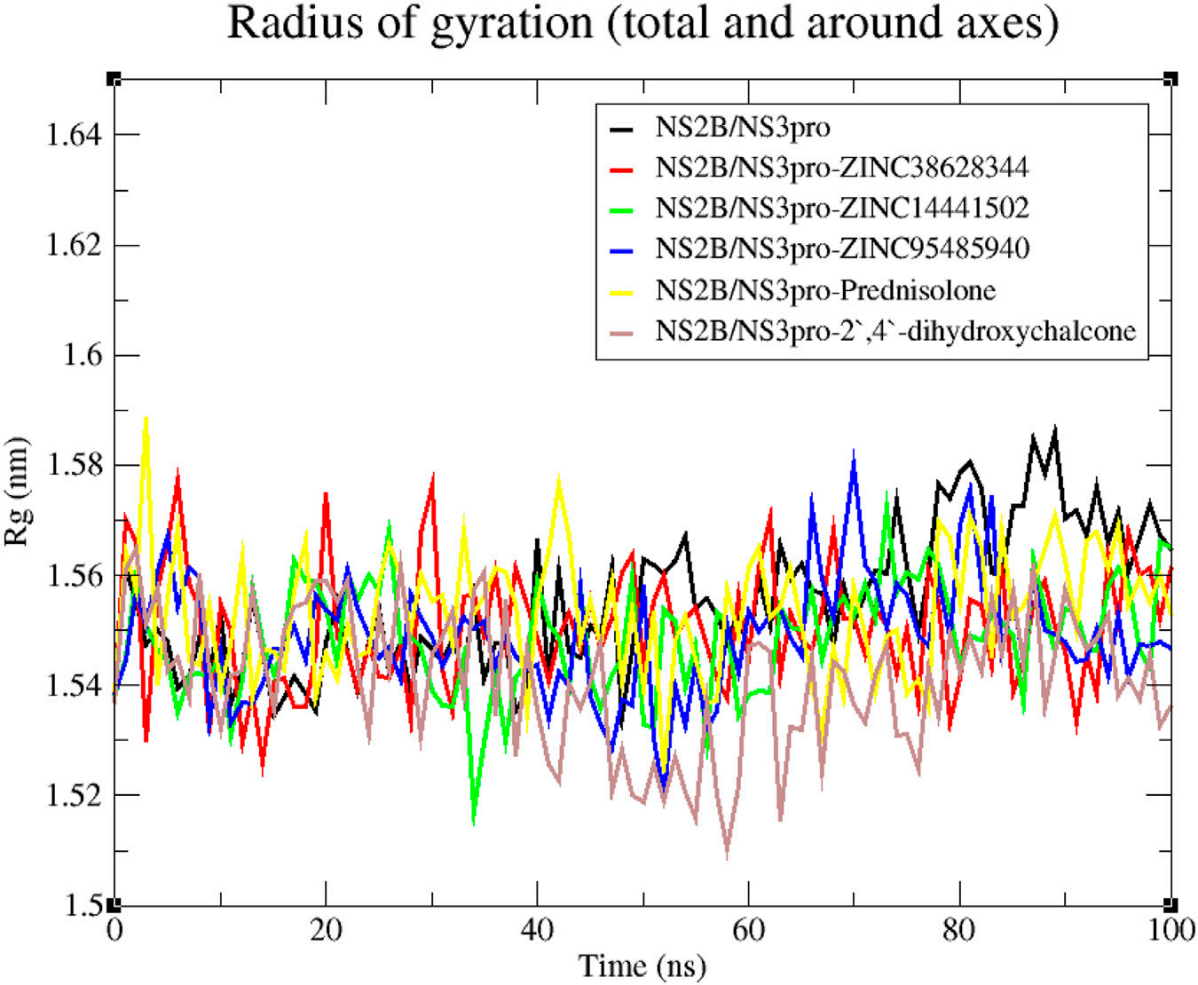
Rg graph of the NS2B/NS3pro-ligand complexes and unbound protein.

The compactness and folding of the five complexes and the unbound protein were examined in this work by charting the radius of gyration (Rg) throughout a 100 ns simulation duration (Liao et al., 2014). A stably folded protein maintains a reasonably steady Rg throughout the simulation. The Rg of the unbound NS2B/NS3 protease and protein-ligand complexes ranged from 1.51 to 1.59 nm (Figure 7). Considering the unbound protease, it experienced relatively steady fluctuation till the 50 ns mark from which it rose sharply till the simulations ended. For the protein-ligand complexes, they demonstrated similar trends in fluctuation throughout the 100 ns run. The Rg for the NS2B/NS3pro-Prednisolone displayed fluctuations with the highest peak at 1.59 nm (Figure 7). The complex NS2B/NS3pro-2′,4′-dihydroxychalcone had the largest fluctuations compared to their complexes, though most of the fluctuations occurred around 40–80 ns.

#### 3.7.3 Root mean square fluctuations (RMSF) for 100 ns MD simulations

Additionally, the RMSF trajectories of the protein-ligand complexes and unbound NS2B/NS3pro were examined (L. Adams et al., 2023; Ashley et al., 2024). According to Cheng and Ivanov (2012), the RMSF reveals a protein’s flexibility in several domains, some of which are connected to crystallographic B-factors. By using this stability profile analysis, residuals that contribute to the structural fluctuation can be evaluated. Greater variations are implied by higher RMSF values. Greater fluctuations occur in protein areas involved in catalysis and ligand binding (Dong et al., 2018). These protein sequence areas that influence the conformational changes of the complex are primarily responsible for adaptive variation in flexibility (Dong et al., 2018).

**FIGURE 8:**
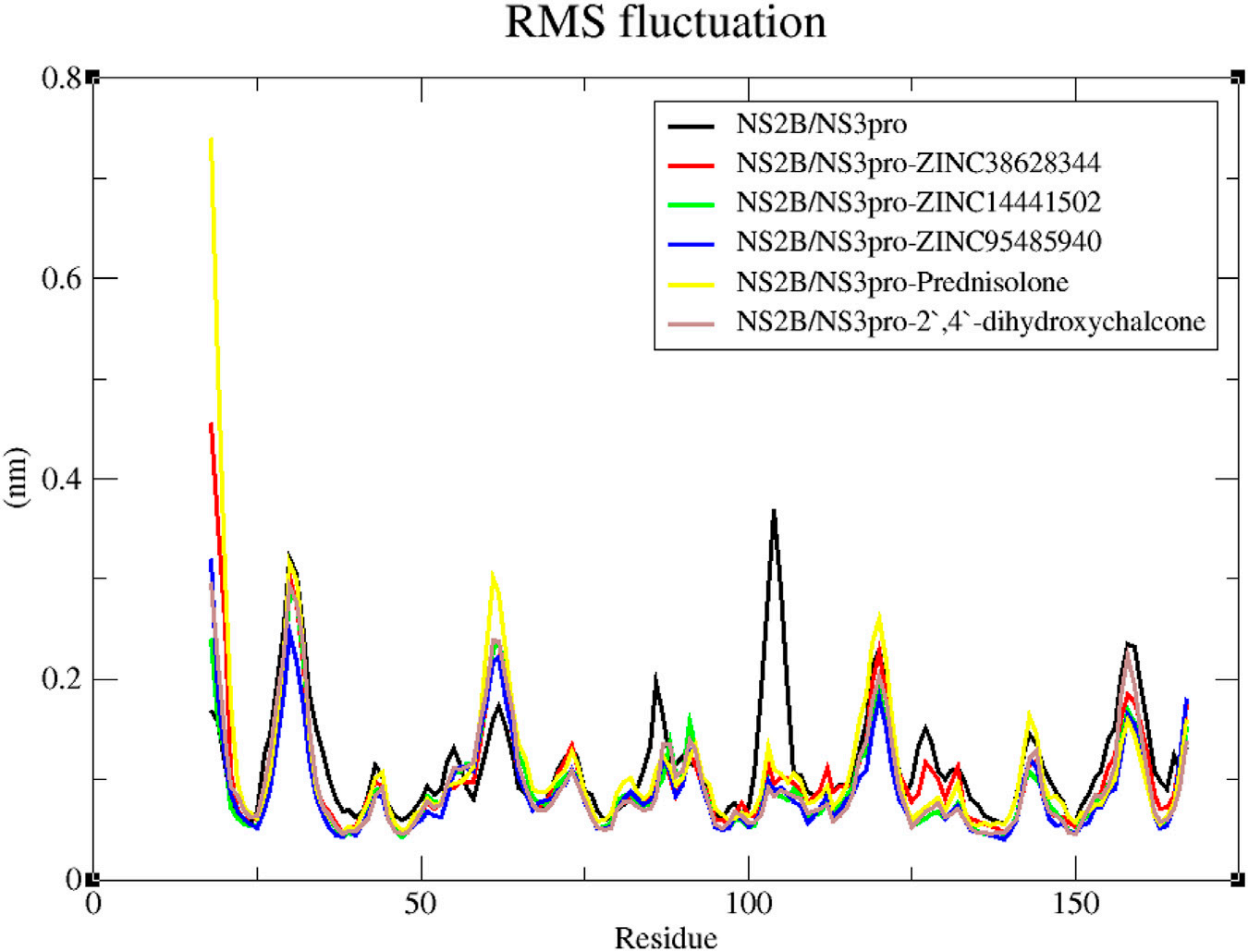
Examination of the RMSF trajectories of the NS2B/NS3pro-ligand complexes and the unbound protein residues.

All the predicted lead compounds caused some degree of changes in comparable regions, according to the RMSF plot (Figure 8). Large fluctuations were observed from residue index 28–33 followed by some fluctuations between residue index 60–65 as well as 116–123. The RMSF graph also showed fluctuations in the unbound protein around residues 102–106 (Figure 8).

### 3.8 MMPBSA computations

#### 3.8.1 Contributing energy terms

The binding free energies of the complexes were estimated using the Molecular Mechanics Poisson-Boltzmann Surface Area (MMPBSA) calculation (Genheden and Ryde, 2015) (Table 5). Van der Waals energies, electrostatic, polar solvation, and solvent-accessible surface area energy are factors that contribute to the binding free energy (Asiedu et al., 2021; Boateng et al., 2020). The binding free energies were computed in terms of average and standard deviations. The leads ZIN38628344, ZINC95485940, and ZINC14441502 and 2′,4′-dihydroxychalcone had binding free energy of −44.957, −18.586, −25.881, and −55.805 kJ/mol respectively. 2′,4′-dihydroxychalcone demonstrated the lowest binding free energy while ZINC95485940 was observed to have the highest binding free energy among the four predicted lead compounds. The binding free energy of the known inhibitor Prednisolone was −17.682 kJ/mol. It has been found that compounds that have high polar energies and low electrostatic energies are active against receptors. (Gupta et al., 2018).

**TABLE 5:** MMPBSA contributing energy terms for NS2B/NS3-ligand complexes displayed as averages ± standard deviations in kJ/mol.

Compounds	van der waal energy (kJ/mol)	Electrostatic energy (kJ/mol)	Polar solvation energy (kJ/mol)	SASA energy (kJ/mol)	Binding energy (kJ/mol)
ZINC38628344	−73.805 ± 4.608	−10.304 ± 1.231	48.041 ± 3.817	−8.983 ± 0.555	−44.957 ± 3.383
ZINC95485940	−54.337 ± 3.716	−65.498 ± 5.335	65.388 ± 4.613	−7.682 ± 0.473	−18.586 ± 2.821
ZINC14441502	−52.459 ± 3.949	−22.090 ± 2.316	41.318 ± 3.042	−6.400 ± 0.476	−25.881 ± 3.519
Prednisolone	−39.913 ± 4.112	−9.190 ± 1.346	36.390 ± 3.989	−5.355 ± 0.527	−17.682 ± 3.583
2′,4′-dihydroxychalcone-(4-O-5‴)-4″,2‴,4‴-trihydroxychalcone	−160.105 ± 5.769	−41.801 ± 2.540	164.633 ± 6.076	−18.440 ± 0.639	−55.805 ± 3.467

#### 3.8.2 Per-residue energy decomposition

By employing per-residue decomposition, the binding energies of individual residues can be computed using the MMPBSA approach. This entails breaking down each residue by taking into account the interactions that each residue participates in. These offer helpful information on significant interactions between crucial residues in the free energy contribution. Critical residues for binding a ligand to a protein contribute binding free energy of at least ±5 kJ/mol (Kwofie et al., 2019a).

**FIGURE 9:**
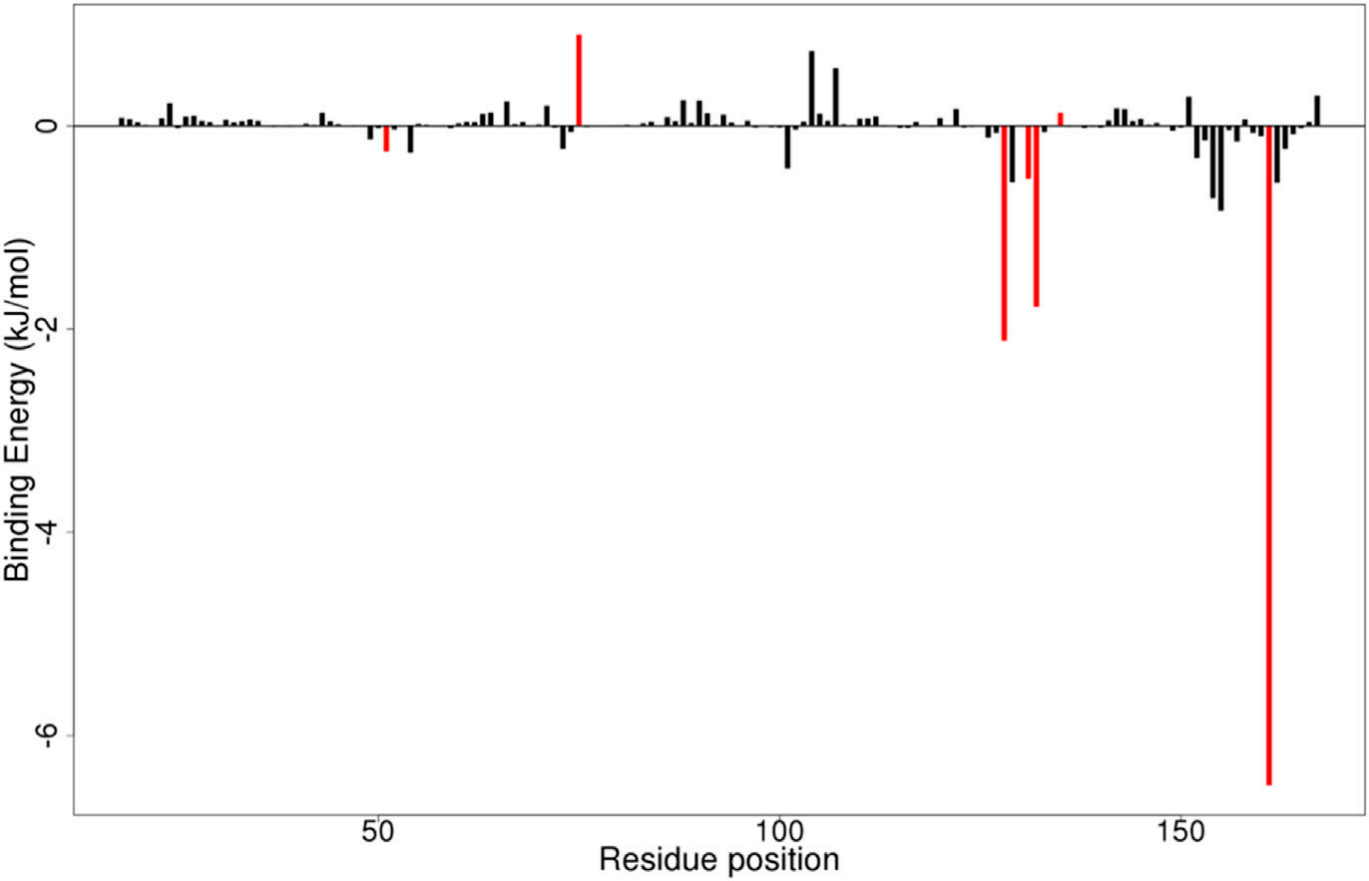
MMPBSA plot of binding free energy contributions per residue for NS2B/NS3-ZINC14441502 complex.

For every complex, the per-residue energy decomposition computation was carried out (Figure 9; Supplementary Figures S1A-D). For the NS2B/NS3-ZINC14441502 complex, only Tyr161 contributed energy of −6.4629 kJ/mol (Figure 9). For the NS2/NS3B-ZINC38628344 complex, Tyr161 and Leu128 contributed individual energies of −6.6957 and −3.4011 kJ/mol respectively (Supplementary Figure S1A). Key residues interacting with ZINC95485940, 2′,4′-dihydroxychalcone, and Prednisolone contributed minor energies (Supplementary Figure 1).

## 4 Discussion

Despite being one of the leading causes of morbidity and mortality in different countries across the world, there is no approved efficacious drug for DENV infection (Palanichamy Kala et al., 2023; Bhatt et al., 2013). While there are currently no approved antiviral drugs or reliable vaccines, a vast number of plants have been tested against DENV through computational discovery (Malabadi et al., 2018; Powers and Setzer, 2016; Rasool et al., 2019; S et al., 2021). The effect of disrupting the function of NS2B/NS3 protease has already been established, particularly through interactions with critical residues His51, Asp75, and Ser135 (Purohit et al., 2022; Tian et al., 2018). In this study, we employed machine learning (ML) and molecular docking techniques to predict potential inhibitors against Dengue Virus (DENV). The combination of machine learning models, molecular dynamics (MD) simulations, and molecular docking enables the exploration of drug candidates and accelerates the identification of lead compounds (Deo, 2015; Niazi and Mariam, 2023; Sarma et al., 2020).

The bioactive dataset from PubChem revealed a significant imbalance, with a ratio of 1:50 between the active and inactive compounds. This class imbalance phenomenon is commonly and largely observed in many bioinformatics and chemoinformatics studies (Japkowicz and Shah, 2011), which can lead to biased model training if not properly addressed. After processing, the dataset was refined to include 4,470 active compounds and 16,780 inactive compounds, achieving a ratio of approximately 1:4. This truncation was performed not merely due to computational constraints but as part of a deliberate effort to enhance the computational efficiency and balance the representation of active and inactive compounds. One key consideration was that excessively large and imbalanced datasets may introduce noise or over-represent the inactive class, thereby diminishing the model’s ability to identify active compounds accurately.

The application of various machine learning algorithms, including k-NN, Gaussian Naïve Bayes, SVM, Random Forest, and Logistic Regression, highlights the diversity of approaches available for predictive modeling of active compounds in pharmacoinformatics (Leardi, 2001; W. Wu &Herath, 2016). The Logistic Regression model emerged as the most effective of the five tested, achieving the highest accuracy, recall, and F1 scores, which are critical metrics for evaluating classifier performance, particularly in imbalanced datasets (Davis and Goadrich, 2006; Mwanga et al., 2023). The performance of other antiviral ML classification models (Gawriljuk et al., 2021; Gupta and Mohanty, 2021; Sandhu et al., 2022), compared to our models though none of them were trained on the dataset used in this study. The recall of 0.76 shows the number of active compounds classified correctly as active while the F1 score of 0.83 illustrates the overall accuracy of the models by combining precision and recall metrics. The SVM closely followed the performance of the LR with a recall and F1 score of 0.71 and 0.81 respectively. In addition, the accuracy of the LR model at 0.94 was not far off from that of the SVM averaging an accuracy of 0.91. An SVM model (Sandhu et al., 2022) trained on 5692 molecules with inhibitory activity against acetylcholinesterase from the bindingDB database establishing accuracy and F1 score of 0.83 and 0.81, respectively. Validation of the model’s predictive power involved testing against known DENV inhibitors, demonstrating the model’s reliability in predicting active compounds. These predictions expand the pool of potential candidate Dengue Virus inhibitors for further validation.

The NS2B/NS3 protease complex, used in this study is composed of the NS3 protease, which carries out the catalytic activity, and the NS2B cofactor, which stabilizes the active conformation of NS3. A serine protease domain located at the N-terminal of NS3 is structurally responsible for cleaving the viral polyprotein into functional units required for viral replication (Low et al., 2011; Bhatt et al., 2013). Given its central role in viral replication, targeting the NS2B/NS3 complex disrupts a key step in the Dengue virus life cycle making NS2B/NS3 protease a key antiviral target for the development of therapeutic agents against the Dengue virus (Erbel et al., 2006; Norshidah et al., 2023; Bhatt et al., 2013). The selection of the PDB ID 2FOM for docking studies was based on structural quality metrics, underscoring the importance of selecting appropriate protein structures for virtual screening (Wlodaweret al., 2008). The results of the docking simulations indicated that anhydrophlegmacin exhibited superior binding affinity, reinforcing the predictive capabilities of the Logistic Regression model. Applying a high threshold of −8.0 kcal/mol improved selection conditions for further studies on active compounds (Kwofie et al., 2019b). Using a threshold of −8.0 kcal/mol, 56 hit compounds that docked firmly were selected for further analysis.

Characterization of ligand interactions using LigPlot provided insight into the binding mechanisms, which are vital for understanding how compounds can effectively inhibit the target protein (Laskowski and Swindells, 2011). This analysis is critical given that it helps identify key residues that contribute significantly to ligand binding, as a means of guiding future drug design efforts. Therefore, hydrogen and hydrophobic interactions between the shortlisted compounds and the residues in the active site helped to elucidate the top-hit compound that inhibits the activities of the NS2B/NS3 protease. Hydrophobic and hydrogen bonds between the selected compounds and catalytic triad His51-Asp75-Ser135 were the main determinants of good interaction and possible inhibition. A structural similarity check between different NS2B/NS3pro confirms the conserved regions and residues (His51-Asp75-Ser135) on the NS3 protease sequence across all serotypes (Purohit et al., 2022; Wahaab et al., 2022), thus the lead compounds identified in this study can serve as cross-serotype inhibitors. The interactions such as His51, Tyr161, Pro132, Asp75, Gly153, and Ser135 by the compounds in this study are consistent with reported binding interactions through docking (Hariono et al., 2019; Purohit et al., 2022; Rasool et al., 2019). The four lead compounds were superimposed to compare their binding modes, revealing that key residues His51, Asp75, and Tyr161 were consistently present across all four complexes. Notably, the residue Ser135 was observed in three lead compounds, ZINC14441502, ZINC95485940, and ZINC38628344. These findings suggest that the four lead compounds share a similar mechanism of action. In addition, the ADMET helps to predict safe compounds by assessing the pharmacokinetic profiles and toxicity of compounds (Wang C. et al., 2018). The integration of Veber’s rules and other drug-like filters serves to prioritize compounds that are more likely to succeed in preclinical and clinical phases denoted as “druglike compounds” (Veber et al., 2002).

Molecular dynamics simulations serve as a powerful tool to explore the stability of protein-ligand complexes over time (Maricarmen et al., 2016). The stability observed in the RMSD plots correlates with the low binding affinities observed during the molecular docking of the compounds. Lower fluctuations often indicate stronger and more stable interactions (Adinortey et al., 2022). However, the inhibitor Prednisolone was observed to be unstable, with its RMSD peaking at 0.3 nm. The predicted leads, ZINC14441502, 2′,4′-dihydroxychalcone, ZINC95485940, and ZINC38628344 demonstrated high fluctuations compared to the unbound state indicating that binding with compounds may have caused conformation changes in the structure. The Rg analysis further supported this by demonstrating the compactness of the complexes, a key indicator of structural integrity during simulations (Liao et al., 2014). A stable Rg between a range of 1.51–1.59 nm, shows a stably folded complex.

Molecular Mechanics Poisson-Boltzmann Surface Area (MMPBSA) calculations provided detailed insights into the energetic contributions of binding interactions, which are crucial for understanding the thermodynamic viability of the ligand-protein complexes (Genheden and Ryde, 2015). The findings indicate that the leads 2′,4′-dihydroxychalcone, ZINC14441502, ZINC95485940, and ZINC38628344 exhibited the most favorable binding free energies of -55.805, -25.881, and -44.957 kJ/mol, respectively, reinforcing their potential as lead compounds for further development (Abdullah et al., 2023; Saleh & Kamisah, 2021). The lead compounds demonstrated a more negative binding energy to the NS2B/NS3 protease than the inhibitor Prednisolone, which is suggestive of a stronger attraction.

Some limitations associated with the study include; the machine learning model was trained on a dataset heavily imbalanced toward inactive compounds, which could influence the model’s ability to generalize well to external datasets. The results validate the logistic model in handling imbalanced datasets, a common issue in bioinformatics studies. While steps were taken to mitigate this, further work could involve data balancing techniques or increasing the number of active compounds to improve predictive accuracy. Additionally, while the molecular docking results are promising, *in vitro* and *in vivo* validations of these compounds are essential to confirm their inhibitory effects against the Dengue virus. Furthermore, pharmacokinetic profiling, though performed *in silico*, would require experimental validation to ensure the predicted compounds meet safety and efficacy standards in a biological system.

## 5 Conclusion

Natural products used as a reservoir for novel therapeutic agents must be tapped and repurposed as effective DENV inhibitors. The significance of this study lies in applying a comprehensive approach to drug discovery, integrating Machine Learning, Molecular Docking, and Dynamics simulations to identify novel potential DENV inhibitors. From this study, five classification models were developed, from which the best-performing model based on accuracy, F1 score, precision, and specificity was employed to make predictions. With an accuracy and precision of 0.94 and 0.91 respectively, the Logistic Regression outperformed the other models and thus was used to predict potential inhibitors against the protease. Four lead compounds ZINC38628344, ZINC95485940, 2′,4′-dihydroxychalcone, and ZINC14441502 with high binding affinities of −0.85, −0.81, −8.6, and −0.81 kcal/mol respectively, and interactions with the conserved catalytic triad, His51-Asp75-Ser135 in the active site of the NS2B/NS3 protease were discovered. The successful prediction and characterization of binding interactions enhance our understanding of ligand-target dynamics based on natural compounds which can be biosynthesized, paving the way for further experimental validations and *in vitro* drug development. The compounds were predicted to possess pharmacokinetic properties and exhibit characteristics of non-tumorigenicity and non-mutagenicity, based on the physicochemical and toxicological characterization, adjourning them to be safe and drug-like. The probable inhibition of the activities of the NS2B/NS3pro of DENV by the leads was corroborated by 100 ns molecular dynamics simulation involving MMPBSA calculations. The study’s approach provides a foundation for the continued use of computational tools in the fight against viral diseases, suggesting a promising path for rapid drug discovery in the future. In addition, the prospective compounds can be considered suitable anti-DENV scaffolds for fragment-based drug design and, thus, are worthy of further experimental validation.

## Data Availability

The dataset used in this study is available at the PubChem Database with AID 651640 (https://pubchem.ncbi.nlm.nih.gov/bioassay/651640) and the GitHub Repo (https://github.com/omicscodeathon/denguedrug/tree/main/data). The comprehensive pipelines and scripts utilized for the models' implementation as well as data and results for the Molecular Docking and Molecular Dynamics Simulations are available at the GitHub repository at https://github.com/omicscodeathon/denguedrug.

## Abbreviations

3D, Three-dimensional; ADMET, chemical Absorption, Distribution, Metabolism, Excretion, and Toxicity; AI, Artificial Intelligence; AID, BioAssay Identification number; ANPDB, African Natural Products Database; DENV, Dengue Virus; DF, Dengue Fever; DHF, Dengue Hemorrhagic Fever; DSS, Dengue Shock Syndrome; EANPDB, Eastern Africa Natural Products Database; GaussianNB, Gaussian Naïve Bayes; GBM, Gradient Boosting Machine; GROMACS, Groningen Machine for Chemical Simulations; HTS, High Throughput Screen; KNN, K-Nearest Neighbors; LR, Logistic Regression; MD, Molecular Dynamics; ML, Machine Learning; MMPBSA, Molecular Mechanics Poisson-Boltzmann Surface Area; NB, Gaussian Naïve Bayes; PaDEL, Pharmaceutical Data Exploration Laboratory; QSAR, Quantitative Structure-Activity Relationship; RF, Random Forest classifier; RMSD, Root Mean Square Deviation; RMSF, Root Mean Square Fluctuation; SASA, Solvent-Accessible Surface Area; SDF, Structure Data Format; SMILES, Simplified Molecular-Input Line-Entry System; SVM, Support Vector Machine; UFF, Universal Force Field.

## References

[B1] Abdullah Z. L. Chee H.-Y. Yusof R. &ohdFauzi F. (2023). Finding lead compounds for dengue antivirals from a collection of old drugs through *in silico* target prediction and subsequent *in vitro* validation. ACS Omega 8 (36), 32483–32497. 10.1021/acsomega.3c02607 37720780 PMC10500654

[B2] Abolo L. Ssenkaali J. Mulumba O. Awe O. I. (2024). Exploring the causal effect of omega-3 polyunsaturated fatty acid levels on the risk of type 1 diabetes: a Mendelian randomization study. Front. Genet. 15, 1353081. 10.3389/fgene.2024.1353081 39040994 PMC11260775

[B3] Abraham M. J. Murtola T. Schulz R. Pall S. Smith J. C. Hess B. (2015). Gromacs: high performance molecular simulations through multi-level parallelism from laptops to supercomputers. SoftwareX 1–2, 19–25. 10.1016/j.softx.2015.06.001

[B4] Adams J. Agyenkwa-mawuli K. Agyapong O. Wilson M. D. &Kwofie S. K. (2022). EBOLApred: a machine learning-based web application for predicting cell entry inhibitors of the Ebola virus. Comput. Biol. Chem. 101 (March), 107766. 10.1016/j.compbiolchem.2022.107766 36088668

[B5] Adams L. Afiadenyo M. Kwofie S. K. Wilson M. D. Kusi K. A. Obiri-Yeboah D. (2023). *In silico* screening of phytochemicals from dissotisrotundifolia against plasmodium falciparum dihydrofolate reductase. Phytomedicine Plus 3 (2), 100447. 10.1016/j.phyplu.2023.100447

[B6] Adinortey C. A. Kwarko G. B. Koranteng R. Boison D. Obuaba I. Wilson M. D. (2022). Molecular structure-based screening of the constituents of calotropis procera identifies potential inhibitors of diabetes mellitus target alpha glucosidase. Curr. Issues Mol. Biol. 44 (2), 963–987. 10.3390/cimb44020064 35723349 PMC8928985

[B7] Agyapong O. Asiedu S. O. Kwofie S. K. Miller W. A. Parry C. S. Sowah R. A. (2021). Molecular modelling and *de novo* fragment-based design of potential inhibitors of beta-tubulin gene of Necatoramericanus from natural products. Inf. Med. Unlocked 26 (February), 100734. 10.1016/j.imu.2021.100734 PMC8670734 34912942

[B8] Alaya F. Baraket G. Adediran D. A. Cuttler K. Ajiboye I. Kivumbi M. T. (2024). Multiple sclerosis stages and their differentially expressed genes: a bioinformatics analysis. 10.1101/2024.01.20.576448

[B9] Anasir M. I. Ramanathan B. (2020) Structure-based design of antivirals against envelope glycoprotein of dengue virus, 1–23. 10.3390/v12040367 PMC7232406 32225021

[B10] Andersson V. O. Ferreira Birck M. A. Araujo R. M. (2018) “Towards predicting dengue fever rates using convolutional neural networks and street-level images,” in 2018 international joint conference on neural networks (IJCNN), 1–8. 10.1109/IJCNN.2018.8489567

[B11] Aniceto N. Albuquerque T. S. Bonifácio V. D. B. Guedes R. C. &rtinho N. (2023). Using machine learning and molecular docking to leverage urease inhibition data for virtual screening. Int. J. Mol. Sci. 24 (9), 8180. 10.3390/ijms24098180 37175889 PMC10179503

[B12] Ashley C. N. Broni E. Wood C. M. Okuneye T. Ojukwu M. P. T. Dong Q. (2024). Identifying potential monkeypox virus inhibitors: an *in silico* study targeting the A42R protein. Front. Cell. Infect. Microbiol. 14 (March), 1351737–1351819. 10.3389/fcimb.2024.1351737 38500508 PMC10945028

[B13] Asiedu S. O. Kwofie S. K. Broni E. Wilson M. D. (2021). Computational identification of potential anti-inflammatory natural compounds targeting the p38 mitogen-activated protein kinase (Mapk): implications for Covid-19-induced cytokine storm. Biomolecules 11 (5), 653. 10.3390/biom11050653 33946644 PMC8146027

[B14] Ather S. H. Awe O. I. Butler T. J. Denka T. Semick S. A. Tang W. (2018). SeqAcademy: an educational pipeline for RNA-Seq and ChIP-Seq analysis. F1000Research 7, 628. 10.12688/f1000research.14880.4 PMC7525341 33014338

[B15] Awe O. I. EnNajih N. Nyamari M. N. Mukanga L. B. (2023). Comparative study between molecular and genetic evolutionary analysis tools using African SARS-CoV2 variants. Inf. Med. Unlocked 36, 101143. 10.1016/j.imu.2022.101143

[B16] Ben A. H. Abassi N. Awe O. I. (2024). NeuroVar: an open-source tool for the visualization of gene expression and variation data for biomarkers of neurological diseases. Gigabyte 2024, gigabyte143. 10.46471/gigabyte.143 39629064 PMC11612633

[B17] Bhatt P. Gething P. W. Brady O. J. Messina J. P. Farlow A. W. Moyes C. L. (2013). The global distribution and burden of dengue. Nature 496 (7446), 504–507. 10.1038/nature12060 23563266 PMC3651993

[B18] Biswal S. Borja-Tabora C. Martinez Vargas L. Velásquez H. Theresa Alera M. Sierra V. (2020). Efficacy of a tetravalent dengue vaccine in healthy children aged 4–16 years: a randomised, placebo-controlled, phase 3 trial. Lancet, 395(10234), 1423–1433. 10.1016/S0140-6736(20)30414-1 32197105

[B19] Boateng R. A. Tastan Bishop Ö. &usyoka T. M. (2020). Characterisation of plasmodial transketolases and identification of potential inhibitors: an *in silico* study. Malar. J. 19 (1), 442–519. 10.1186/s12936-020-03512-1 33256744 PMC7756947

[B20] Byrd C. M. Dai D. Grosenbach D. W. Berhanu A. Jones K. F. Cardwell K. B. (2013). A novel inhibitor of dengue virus replication that targets the capsid protein. Antimicrob. Agents Chemother. 57 (1), 15–25. 10.1128/AAC.01429-12 23070172 PMC3535982

[B21] Caminade C. Medlock J. M. Ducheyne E. Mcintyre K. M. Leach S. Baylis M. (2012). Suitability of European climate for the Asian tiger mosquito *Aedes albopictus*: recent trends and future scenarios, 2708–2717. 10.1098/rsif.2012.0138 PMC3427500 22535696

[B22] Carocci M. Hinshaw S. M. Rodgers M. A. Villareal V. A. Burri D. J. Pilankatta R. (2015). The bioactive lipid 4-hydroxyphenyl retinamide inhibits Flavivirus replication. Antimicrob. Agents Chemother. 59 (1), 85–95. 10.1128/AAC.04177-14 25313218 PMC4291433

[B23] Chao L. H. Jang J. Johnson A. Nguyen A. Gray N. S. Yang P. L. (2018) How small-molecule inhibitors of dengue-virus infection interfere with viral membrane fusion, 1–11. 10.7554/eLife.36461 PMC6056230 29999491

[B24] Che P. Wang L. Li Q. (2009). The development, optimization and validation of an assay for high throughput antiviral drug screening against dengue virus. Int. J. Clin. Exp. Med. 2 (4), 363–373. 20057980 PMC2802053

[B25] Chen H.-R. Lai Y.-C. Yeh T.-M. (2018). Dengue virus non-structural protein 1: a pathogenic factor, therapeutic target, and vaccine candidate. J. Biomed. Sci. 25 (1), 58–11. 10.1186/S12929-018-0462-0 30037331 PMC6057007

[B144] Cheng X. Ivanov I. (2012). Molecular dynamics. Methods Mol. Biol. (Clifton, N.J.) 929, 243–285. 10.1007/978-1-62703-050-2_11 23007433

[B26] Chikkaveeraiah S. K. Srinath K. M. Hathur B. V. Hari V. S. A. &Vanama L. S. (2024). Study of dengue fever in an epidemic - a single centre observational study at tertiary care hospital. APIK J. Intern. Med. 12 (4), 205–209. 10.4103/ajim.ajim_45_23

[B27] Chikwambi Z. Hidjo M. Chikondowa P. Afolabi L. Aketch V. Jayeoba G. (2023). Multi-omics data integration approach identifies potential biomarkers for Prostate cancer. bioRxiv. 10.1101/2023.01.26.522643

[B28] Ci Y. Yao B. Yue K. Yang Y. Xu C. Li D.-F. (2023). Bortezomib inhibits ZIKV/DENV by interfering with viral polyprotein cleavage via the ERAD pathway. Cell. Chem. Biol. 30 (5), 527–539.e5. 10.1016/j.chembiol.2022.10.003 36351431

[B29] Comesana A. E. Huntington T. T. Scown C. D. Niemeyer K. E. Rapp V. H. (2022). A systematic method for selecting molecular descriptors as features when training models for predicting physiochemical properties. Fuel, 321, 123836. 10.1016/j.fuel.2022.123836

[B30] Cucunawangsih L. N. Lugito N. P. H. (2017). Trends of dengue disease epidemiology. Virology Res. Treat. 8. 10.1177/1178122X17695836 PMC5428083 28579763

[B31] Daina A. Michielin O. &Zoete V. (2017). SwissADME: a free web tool to evaluate pharmacokinetics, drug-likeness and medicinal chemistry friendliness of small molecules. Sci. Rep. 7 (January), 42717–42813. 10.1038/srep42717 28256516 PMC5335600

[B32] Das A. P. Mathur P. Agarwal S. M. (2024). Machine learning, molecular docking, and dynamics-based computational identification of potential inhibitors against lung cancer. Mach. Learn. Mol. Docking, Dynamics-Based Comput. Identif. Potential Inhibitors against Lung Cancer 9, 4528–4539. 10.1021/acsomega.3c07338 PMC10831845 38313551

[B33] Davis J. Goadrich M. (2006) “The relationship between Precision-Recall and ROC curves,” in Proceedings of the 23rd international conference on machine learning, 233–240. 10.1145/1143844.1143874

[B34] Deo R. C. (2015). Machine learning in medicine. Circulation 132 (20), 1920–1930. 10.1161/CIRCULATIONAHA.115.001593 26572668 PMC5831252

[B35] Devadasu V. R. Deb P. K. Maheshwari R. Sharma P. &Tekade R. K. (2018) “Physicochemical, pharmaceutical, and biological considerations in GIT absorption of drugs,” in Dosage form design considerations, I. Elsevier Inc, 149–178. 10.1016/B978-0-12-814423-7.00005-8

[B36] de Wispelaere M. LaCroix A. J. Yang P. L. (2013). The small molecules AZD0530 and dasatinib inhibit dengue virus RNA replication via Fyn kinase. J. Virology 87 (13), 7367–7381. 10.1128/JVI.00632-13 23616652 PMC3700292

[B37] Die J. V. Elmassry M. M. LeBlanc K. H. Awe O. I. Dillman A. Busby B. (2019). geneHummus: an R package to define gene families and their expression in legumes and beyond. BMC Genomics 20, 591. 10.1186/s12864-019-5952-2 31319791 PMC6639926

[B38] Dong Y. W. Liao M. L. Meng X. L. &Somero G. N. (2018). Structural flexibility and protein adaptation to temperature: molecular dynamics analysis of malate dehydrogenases of marine molluscs. Proc. Natl. Acad. Sci. U. S. A. 115 (6), 1274–1279. 10.1073/pnas.1718910115 29358381 PMC5819447

[B39] Drago F. Ciccarese G. Merlo G. Trave I. Javor S. Rebora A. (2021). Oral and cutaneous manifestations of viral and bacterial infections: not only COVID-19 disease. Clin. Dermatology 39 (3), 384–404. 10.1016/j.clindermatol.2021.01.021 PMC7849469 34517997

[B40] Durbin A. P. (2020). Historical discourse on the development of the live attenuated tetravalent dengue vaccine candidate TV003/TV005. Curr. Opin. Virology, 43, 79–87. 10.1016/j.coviro.2020.09.005 PMC7685199 33164790

[B41] Dwivedi V. D. Tripathi I. P. Tripathi R. C. Bharadwaj S. Mishra S. K. (2017). Genomics, proteomics and evolution of dengue virus. Briefings Funct. Genomics 16 (4), 217–227. 10.1093/bfgp/elw040 28073742

[B42] El Abed F. Baraket G. Nyamari M. N. Naitore C. Awe O. I. (2023). Differential expression analysis of miRNAs and mRNAs in epilepsy uncovers potential biomarkers. bioRxiv. 10.1101/2023.09.11.557132

[B43] Erbel P. Schiering N. D’Arcy A. Renatus M. Kroemer M. Lim S. P. (2006). Structural basis for the activation of flaviviral NS3 proteases from dengue and West Nile virus. Nat. Struct. Mol. Biol. 13 (4), 372–373. 10.1038/nsmb1073 16532006

[B44] Franco E. J. de Mello C. P. P. Brown A. N. (2021). Antiviral evaluation of uv-4b and interferon-alpha combination regimens against dengue virus. Viruses 13 (5), 771. 10.3390/v13050771 33925551 PMC8145572

[B45] Fraser J. E. Watanabe S. Wang C. Chan W. K. K. Maher B. Lopez-Denman A. (2014). A nuclear transport inhibitor that modulates the unfolded protein response and provides *in vivo* protection against lethal dengue virus infection. J. Infect. Dis. 210 (11), 1780–1791. 10.1093/infdis/jiu319 24903662

[B46] Gautam S. Thakur A. Rajput A. Kumar M. (2024). Anti-dengue: a machine learning-assisted prediction of small molecule antivirals against dengue virus and implications in drug repurposing. Viruses 16 (1), 45. 10.3390/v16010045 PMC10818795 38257744

[B47] Gawriljuk V. O. Zin P. P. K. Puhl A. C. Zorn K. M. Foil D. H. Lane T. R. (2021). Machine learning models identify inhibitors of SARS-CoV-2. J. Chem. Inf. Model. 61 (9), 4224–4235. 10.1021/acs.jcim.1c00683 34387990 PMC8574161

[B48] Gebhard L. G. Leal E. S. Adler N. S. Fern G. A. Battini L. Aucar M. G. (2019) European Journal of Medicinal Chemistry De novo design approaches targeting an envelope protein pocket to identify small molecules against dengue virus, 182. 10.1016/j.ejmech.2019.111628 31472473

[B49] Genheden S. Ryde U. (2015). The MM/PBSA and MM/GBSA methods to estimate ligand-binding affinities. Expert Opin. Drug Discov. 10, 449–461. 10.1517/17460441.2015.1032936 25835573 PMC4487606

[B50] Gupta A. Chaudhary N. &roy P. (2018). MM-PBSA and per-residue decomposition energy studies on 7-Phenyl-imidazoquinolin-4(5H)-one derivatives: identification of crucial site points at microsomal prostaglandin E synthase-1 (mPGES-1) active site. Int. J. Biol. Macromol. 119, 352–359. 10.1016/j.ijbiomac.2018.07.050 30031079

[B51] Gupta P. Mohanty D. (2021). SMMPPI: a machine learning-based approach for prediction of modulators of protein-protein interactions and its application for identification of novel inhibitors for RBD:hACE2 interactions in SARS-CoV-2. Briefings Bioinforma. 22 (5), bbab111–15. 10.1093/bib/bbab111 PMC8083326 33839740

[B52] Hariono M. Choi S. B. Roslim R. F. Nawi M. S. Tan M. L. Kamarulzaman E. E. (2019). Thioguanine-based DENV-2 NS2B/NS3 protease inhibitors: virtual screening, synthesis, biological evaluation and molecular modelling. PLoS ONE 14 (1), 02108699–e210921. 10.1371/journal.pone.0210869 PMC6345492 30677071

[B53] Jamal Z. Haider S. A. Hakim R. Humayun F. Umar M. Muhammad F. (2024). Serotype and genomic diversity of dengue virus during the 2023 outbreak in Pakistan reveals the circulation of genotype III of DENV‐1 and cosmopolitan genotype of DENV‐2. J. Med. Virol. 96, 297277–e29813. 10.1002/jmv.29727 38864343

[B54] Japkowicz N. Shah M. (2011). Evaluating learning algorithms: a classification perspective. Cambridge University Press.

[B55] Jia C. Y. Li J. Y. Hao G. F. Yang G. F. (2020). A drug-likeness toolbox facilitates ADMET study in drug discovery. Drug Discov. Today 25 (1), 248–258. 10.1016/j.drudis.2019.10.014 31705979

[B57] Jung Y. Hu J. (2015). A K-fold averaging cross-validation procedure. J. Nonparametric Statistics 27 (2), 167–179. 10.1080/10485252.2015.1010532 PMC5019184 27630515

[B58] Kallas E. G. Precioso A. R. Palacios R. Thomé B. Braga P. E. Vanni T. (2020). Safety and immunogenicity of the tetravalent, live-attenuated dengue vaccine Butantan-DV in adults in Brazil: a two-step, double-blind, randomised placebo-controlled phase 2 trial. Lancet Infect. Dis., 20(7), 839–850. 10.1016/S1473-3099(20)30023-2 32220283

[B59] Kee E. Chong J. J. Choong Z. J. Lau M. (2023). A comparative analysis of cross-validation techniques for a smart and lean pick-and-place solution with deep learning. Electron. Switz. 12 (11), 2371. 10.3390/electronics12112371

[B60] Khorshid S. F. Abdulazeez A. M. Sallow A. B. (2021). A comparative analysis and predicting for breast cancer detection based on data mining models. Asian J. Res. Comput. Sci. May, 45–59. 10.9734/ajrcos/2021/v8i430209

[B61] Kularatne S. A. M. Walathara C. Mahindawansa S. I. Wijesinghe S. Pathirage M. M. K. Kumarasiri P. V. R. (2009). Efficacy of low dose dexamethasone in severe thrombocytopenia caused by dengue fever: a placebo controlled study. Postgrad. Med. J. 85 (1008), 525–529. 10.1136/pgmj.2008.078444 19789191

[B62] Kumari R. Kumar R. Lynn A. (2014). *g_mmpbsa*—a Gromacs Tool for High-Throughput MM-PBSA Calculations. J. Chem. Inf. Model. 54, 1951–1962. 10.1021/ci500020m 24850022

[B63] Kwofie S. K. Broni E. Teye J. Quansah E. Issah I. Wilson M. D. (2019a). Pharmacoinformatics-based identification of potential bioactive compounds against Ebola virus protein VP24. Comput. Biol. Med. 113 (March), 103414. 10.1016/j.compbiomed.2019.103414 31536833

[B64] Kwofie S. K. Dankwa B. Enninful K. S. Adobor C. Broni E. Ntiamoah A. (2019b). Molecular docking and dynamics simulation studies predict munc18b as a target of mycolactone: a plausible mechanism for granule exocytosis impairment in Buruli Ulcer Pathogenesis. Toxins 11 (3), 181. 10.3390/toxins11030181 30934618 PMC6468854

[B65] Kwofie S. K. Hanson G. Sasu H. Enninful K. S. Mensah F. A. Nortey R. T. (2022). Molecular modelling and atomistic insights into the binding mechanism of MmpL3 mtb. Chem. Biodivers. 19 (9), e202200160. 10.1002/cbdv.202200160 35969844

[B66] Lai J.-H. Lin Y.-L. Hsieh S.-L. (2017). Pharmacological intervention for dengue virus infection. Biochem. Pharmacol. 129, 14–25. 10.1016/j.bcp.2017.01.005 28104437

[B67] Laskowski R. A. Swindells M. B. (2011). LigPlot+: multiple ligand–protein interaction diagrams for drug discovery. J. Chem. Inf. Model. 51 (10), 2778–2786. 10.1021/ci200227u 21919503

[B68] Leardi R. (2001). Genetic algorithms in chemometrics and chemistry: a review. J. Chemom., 15(7), 559–569. 10.1002/cem.651

[B69] Lee J.-C. Tseng C.-K. Wu Y.-H. Kaushik-Basu N. Lin C.-K. Chen W.-C. (2015). Characterization of the activity of 2’-C-methylcytidine against dengue virus replication. Antivir. Res. 116, 1–9. 10.1016/j.antiviral.2015.01.002 25614455

[B70] Lee M. F. Tan S. L. Ahemad N. Hamid A. A. A. Hishamuddin S. A. S. N. Batumalaie K. (2024). Molecular docking and dynamics simulation reveal withanolides as potent antivirals against dengue virus. South Afr. J. Bot., 169, 426–434. 10.1016/j.sajb.2024.04.045

[B71] Liao K. H. Chen K. B. Lee W. Y. Sun M. F. Lee C. C. Chen C. Y. C. (2014). Ligand-based and structure-based investigation for Alzheimer’s disease from traditional Chinese medicine. Evidence-Based Complementary Altern. Med. 2014, 364819. 10.1155/2014/364819 PMC4034731 24899907

[B72] Lim S. P. (2019). Dengue drug discovery: progress, challenges and outlook. Antivir. Res. 163, 156–178. 10.1016/J.ANTIVIRAL.2018.12.016 30597183

[B73] Lim X.-N. Shan C. Marzinek J. K. Dong H. Ng T. S. Ooi J. S. G. (2019). Molecular basis of dengue virus serotype 2 morphological switch from 29°C to 37°C. PLoS Pathog. 15 (9), e1007996. 10.1371/journal.ppat.1007996 31536610 PMC6752767

[B74] Lin C. L. Kiu Y. T. Kan J. Y. Chang Y. J. Hung P. Y. Lu C. H. (2023). The antiviral activity of varenicline against dengue virus replication during the post-entry stage. Biomedicines 11 (10), 2754–2814. 10.3390/biomedicines11102754 37893127 PMC10604274

[B75] Low J. S. Y. Wu K. X. Chen K. C. Ng M. M. L. Chu J. J. H. (2011). Narasin, a novel antiviral compound that blocks dengue virus protein expression. Antivir. Ther. 16 (8), 1203–1218. 10.3851/IMP1884 22155902

[B76] Malabadi R. B. Chalannavar R. K. Supriya S. Nityasree B. R. Sowmyashree K. &Meti N. T. (2018). Role of botanical drugs in controlling dengue virus disease. Available at: https://api.semanticscholar.org/CorpusID:212505980.

[B77] Maricarmen H.-R. Martha R.-H. C. Jessica M.-W. E. Marlet M.-A. José B. C. (2016). Current tools and methods in molecular dynamics (MD) simulations for drug design. In Curr. Med. Chem. (Vol. 23, Issue 34, pp. 3909–3924). 10.2174/0929867323666160530144742 27237821

[B78] Martina B. E. E. Koraka P. Osterhaus A. D. M. E. (2009). Dengue virus pathogenesis: an integrated view. Clin. Microbiol. Rev. 22 (4), 564–581. 10.1128/CMR.00035-09 19822889 PMC2772360

[B79] Mazumder M. Ponnan P. Das U. Gourinath S. Khan H. A. Yang J. (2017). Investigations on binding pattern of kinase inhibitors with PPAR γ: molecular docking, molecular dynamic simulations, and free energy calculation studies. PPAR Res. 2017, 1–11. 10.1155/2017/6397836 PMC5340984 28321247

[B80] McCormick K. D. Liu S. Jacobs J. L. Marques E. T. A. Sluis-Cremer N. Wang T. (2012). Development of a robust cytopathic effect-based high-throughput screening assay to identify novel inhibitors of dengue virus. Antimicrob. Agents Chemother. 56 (6), 3399–3401. 10.1128/AAC.06425-11 22391547 PMC3370735

[B81] Murray N. E. A. Quam M. B. Wilder-Smith A. (2013). Epidemiology of dengue: past, present and future prospects. Clin. Epidemiol. 5 (1), 299–309. 10.2147/CLEP.S34440 23990732 PMC3753061

[B82] Musyoka T. M. Kanzi A. M. Lobb K. A. &Tastan Bishop Ö. (2016). Structure based docking and molecular dynamic studies of plasmodial cysteine proteases against a South African natural compound and its analogs. Sci. Rep. 6 (December 2015), 23690–23712. 10.1038/srep23690 27030511 PMC4814779

[B83] Mwanga M. J. Obura H. Evans M. Awe O. I. (2023). Enhanced deep convolutional neural Network for SARS-CoV-2 variants classification. BioRxiv, 2023–2028. 10.1101/2023.08.09.552643 PMC12450893 40988919

[B84] Nguyen N. M. Tran C. N. B. Phung L. K. Duong K. T. H. Huynh H. le A. Farrar J. (2013). A randomized, double-blind placebo controlled trial of balapiravir, a polymerase inhibitor, in adult dengue patients. J. Infect. Dis. 207 (9), 1442–1450. 10.1093/infdis/jis470 22807519 PMC3610419

[B85] Niazi S. K. Mariam Z. (2023). Recent advances in machine-learning-based chemoinformatics: a comprehensive review. Int. J. Mol. Sci. 24 (14), 11488. 10.3390/ijms241411488 37511247 PMC10380192

[B86] Niazi S. K. Mariam Z. (2024) Computer-aided drug design and drug discovery: a prospective analysis, 1–22. 10.3390/ph17010022 PMC10819513 38256856

[B87] Noble C. G. Seh C. C. Chao A. T. Shi P. Y. (2012). Ligand-bound structures of the dengue virus protease reveal the active conformation. J. Virology 86 (1), 438–446. 10.1128/JVI.06225-11 22031935 PMC3255909

[B88] Norshidah H. Leow C. H. Ezleen K. E. Wahab H. A. Vignesh R. Rasul A. (2023). Assessing the potential of NS2B/NS3 protease inhibitors biomarker in curbing dengue virus infections: *in silico* vs. *in vitro* approach. Front. Cell. Infect. Microbiol. 13 (February), 1061937–1062017. 10.3389/fcimb.2023.1061937 36864886 PMC9971573

[B89] Ntie-kang F. Zofou D. Babiaka S. B. Meudom R. Scharfe M. Lifongo L. L. (2013). AfroDb: a select highly potent and diverse natural product library from african medicinal plants. PLoS One 8 (10), 780855–e78115. 10.1371/journal.pone.0078085 PMC3813505 24205103

[B90] Nyamari M. N. Omar K. M. Fayehun A. F. Dachi O. Bwana B. K. Awe O. I. (2023). Expression level analysis of ACE2 receptor gene in african-American and non-african-American COVID-19 patients. bioRxiv. 10.1101/2023.09.11.557129

[B91] Nzungize L. Kengne-Ouafo J. A. Wesonga M. R. Umuhoza D. Murithi K. Kimani P. (2022). Transcriptional profiles analysis of COVID-19 and malaria patients reveals potential biomarkers in children. bioRxiv. 10.1101/2022.06.30.498338

[B92] O’Boyle N. M. Banck M. James C. A. Morley C. Vandermeersch T. Hutchison G. R. (2011). Open Babel: an open chemical toolbox. J. Cheminformatics 3, 33. 10.1186/1758-2946-3-33 PMC3198950 21982300

[B93] Obura H. O. Mlay C. D. Moyo L. Karumbo B. M. Omar K. M. Sinza E. M. (2022). Molecular phylogenetics of HIV-1 subtypes in african populations: a case study of sub-saharan african countries. bioRxiv. 10.1101/2022.05.18.492401

[B94] Ogbodo U. C. Enejoh O. A. Okonkwo C. H. Gnanasekar P. Gachanja P. W. Osata S. (2023). Computational identification of potential inhibitors targeting cdk1 in colorectal cancer. Front. Chem. 11 (November), 1264808–1264817. 10.3389/fchem.2023.1264808 38099190 PMC10720044

[B95] Oluwagbemi O. Awe O. I. (2018). A comparative computational genomics of Ebola Virus Disease strains: in-silico Insight for Ebola control. Inf. Med. Unlocked 12, 106–119. 10.1016/j.imu.2018.07.004

[B96] Omar K. M. Kitundu G. L. Jimoh A. O. Namikelwa D. N. Lisso F. M. Babajide A. A. (2024). Investigating antimicrobial resistance genes in Kenya, Uganda and Tanzania cattle using metagenomics. PeerJ 12, e17181. 10.7717/peerj.17181 38666081 PMC11044882

[B97] Orozco-arias S. Piña J. S. Tabares-soto R. Castillo-ossa L. F. Guyot R. Isaza G. (2020). Measuring performance metrics of machine learning algorithms for detecting and classifying transposable elements. May 8, 638. 10.3390/pr8060638

[B98] Palanichamy Kala M. St. John A. L. Rathore A. P. S. (2023). Dengue: update on clinically relevant therapeutic strategies and vaccines. Curr. Treat. Options Infect. Dis. 15 (2), 27–52. 10.1007/s40506-023-00263-w 37124673 PMC10111087

[B99] Pawar S. V. Banini W. S. K. Shamsuddeen M. M. Jumah T. A. Dolling N. N. O. Tiamiyu A. (2024). Proestrus: an open-source tool for 3D structure prediction using homology modeling. Front. Chem. 12, 1509407. 10.3389/fchem.2024.1509407 39717221 PMC11664737

[B100] Pedregosa F. Varoquaux G. Gramfort A. Michel V. Thirion B. Grisel O. (2011). Scikit-learn: machine learning in Python. J. Mach. Learn. Res. 12 (85), 2825–2830. Available at: http://jmlr.org/papers/v12/pedregosa11a.html.

[B101] Pintado Silva J. Fernandez-Sesma A. (2023). Challenges on the development of a dengue vaccine: a comprehensive review of the state of the art. J. General Virology 104 (3), 001831. 10.1099/jgv.0.001831 PMC10228381 36857199

[B102] Powers C. Setzer N. (2016). An in-silico investigation of phytochemicals as antiviral agents against dengue fever. Comb. Chem. and High Throughput Screen. 19 (7), 516–536. 10.2174/1386207319666160506123715 27151482 PMC5411999

[B103] Punekar M. Kasabe B. Patil P. Kakade M. B. Parashar D. Alagarasu K. (2022). A transcriptomics-based bioinformatics approach for identification and *in vitro* screening of FDA-approved drugs for repurposing against dengue virus-2. Viruses 14 (10), 2150. 10.3390/v14102150 36298705 PMC9609047

[B104] Purohit P. Sahoo S. Panda M. Sahoo P. S. &eher B. R. (2022). Targeting the DENV NS2B-NS3 protease with active antiviral phytocompounds: structure-based virtual screening, molecular docking and molecular dynamics simulation studies. J. Mol. Model. 28 (11), 365. 10.1007/s00894-022-05355-w 36274116 PMC9589672

[B105] Rachmawati Y. Ekawardhani S. Fauziah N. Faridah L. Watanabe K. (2024). Potential way to develop dengue virus detection in Aedes larvae as an alternative for dengue active surveillance: a literature review. Trop. Med. Infect. Dis. 9 (3), 60. 10.3390/tropicalmed9030060 38535884 PMC10975107

[B106] Raekiansyah M. Mori M. Nonaka K. Agoh M. Shiomi K. Matsumoto A. (2017). Identification of novel antiviral of fungus-derived brefeldin A against dengue viruses. Trop. Med. Health 45 (1), 32–37. 10.1186/s41182-017-0072-7 29093640 PMC5658972

[B107] Rasool N. Ashraf A. Waseem M. Hussain W. Mahmood S. (2019). Computational exploration of antiviral activity of phytochemicals against NS2B/NS3 proteases from dengue virus. , 44(3), 261–277. 10.1515/tjb-2018-0002

[B108] S A. H. Pujar G. V. Sethu A. K. Bhagyalalitha M. Singh M. (2021). Dengue structural proteins as antiviral drug targets: current status in the drug discovery and development. Eur. J. Med. Chem., 221, 113527. 10.1016/j.ejmech.2021.113527 34020338

[B109] Saleh M. S. M. &Kamisah Y. (2021). Potential medicinal plants for the treatment of dengue fever and severe acute respiratory syndrome-coronavirus. Biomolecules 11 (1), 42–25. 10.3390/biom11010042 PMC7824034 33396926

[B110] Salgado D. Zabaleta T. E. Hatch S. Vega M. R. Rodriguez J. (2012). Use of pentoxifylline in treatment of children with dengue hemorrhagic fever. Pediatr. Infect. Dis. J. 31 (7), 771–773. 10.1097/INF.0b013e3182575e6a 22481426

[B111] Sanchez-Gendriz I. de Souza G. F. de Andrade I. G. M. Neto A. D. D. de Medeiros Tavares A. Barros D. M. S. (2022). Data-driven computational intelligence applied to dengue outbreak forecasting: a case study at the scale of the city of Natal, RN-Brazil. Sci. Rep. 12 (1), 6550–6610. 10.1038/s41598-022-10512-5 35449179 PMC9023501

[B112] Sander T. Freyss J. Von Korff M. &Rufener C. (2015). DataWarrior: an open-source program for chemistry aware data visualization and analysis. J. Chem. Inf. Model. 55 (2), 460–473. 10.1021/ci500588j 25558886

[B113] Sandhu H. Kumar R. N. Garg P. (2022). Machine learning-based modeling to predict inhibitors of acetylcholinesterase. Mol. Divers. 26 (1), 331–340. 10.1007/s11030-021-10223-5 33891263

[B114] Sarma D. Hossain S. Mittra T. Bhuiya M. A. M. Saha I. Chakma R. (2020) “Dengue prediction using machine learning algorithms,” in 2020 IEEE 8th R10 humanitarian technology conference (R10-HTC), 1–6. 10.1109/R10-HTC49770.2020.9357035

[B115] Simanjuntak Y. Liang J.-J. Lee Y.-L. Lin Y.-L. (2015). Repurposing of prochlorperazine for use against dengue virus infection. J. Infect. Dis. 211 (3), 394–404. 10.1093/infdis/jiu377 25028694

[B116] Simoben C. V. Qaseem A. Moumbock A. F. A. Telukunta K. K. Günther S. Sippl W. (2020). Pharmacoinformatic investigation of medicinal plants from East Africa. Mol. Inf. 39 (11), 20001633–e2000215. 10.1002/minf.202000163 PMC7685152 32964659

[B117] Smith J. L. Stein D. A. Shum D. Fischer M. A. Radu C. Bhinder B. (2014). Inhibition of dengue virus replication by a class of small-molecule compounds that antagonize dopamine receptor d4 and downstream mitogen-activated protein kinase signaling. J. Virology 88 (10), 5533–5542. 10.1128/JVI.00365-14 24599995 PMC4019099

[B118] Sterling T. Irwin J. J. (2015). ZINC 15 - ligand discovery for everyone. J. Chem. Inf. Model. 55 (11), 2324–2337. 10.1021/acs.jcim.5b00559 26479676 PMC4658288

[B119] Suenderhauf C. Hammann F. &Huwyler J. (2012). Computational prediction of blood-brain barrier permeability using decision tree induction. Molecules 17 (9), 10429–10445. 10.3390/molecules170910429 22941223 PMC6269008

[B120] Tharwat A. (2021). Classification assessment methods. Appl. Comput. Inf. 17 (1), 168–192. 10.1016/j.aci.2018.08.003

[B121] Thomas S. J. (2023). Is new dengue vaccine efficacy data a relief or cause for concern? Npj Vaccines 8 (1), 55. 10.1038/s41541-023-00658-2 37061527 PMC10105158

[B122] Tian Y. S. Zhou Y. Takagi T. Kameoka M. &Kawashita N. (2018). Dengue virus and its inhibitors: a brief review. Chem. Pharm. Bull. 66 (3), 191–206. 10.1248/cpb.c17-00794 29491253

[B123] Tougui I. Jilbab A. &hamdi J.El. (2021). Impact of the choice of cross-validation techniques on the results of machine learning-based diagnostic applications. Healthc. Inf. Res. 27 (3), 189–199. 10.4258/hir.2021.27.3.189 PMC8369053 34384201

[B124] Trinh C. Tbatou Y. Lasala S. Herbinet O. &eimaroglou D. (2023). On the development of descriptor-based machine learning models for thermodynamic properties: Part 1—from data collection to model construction: understanding of the methods and their effects. Processes 11 (12), 3325–3340. 10.3390/pr11123325

[B125] Trott O. Olson A. J. (2010). AutoDock Vina: improving the speed and accuracy of docking with a new scoring function, efficient optimization, and multithreading. J. Comput. Chem. 31 (2), 455–461. 10.1002/jcc.21334 19499576 PMC3041641

[B126] Turner P. (2005). XMGRACE, version 5.1. 19. *Center for Coastal and land-margin research* . Oregon Graduate Institute of Science and Technology, Beaverton. 10.1163/_q3_SIM_00374

[B127] Vabalas A. Gowen E. Poliakoff E. &Casson A. J. (2019). Machine learning algorithm validation with a limited sample size. PLOS ONE 14 (11), e0224365. 10.1371/journal.pone.0224365 31697686 PMC6837442

[B128] Veber D. F. Johnson S. R. Cheng H. Smith B. R. Ward K. W. Kopple K. D. (2002). Molecular properties that influence the oral bioavailability of drug candidates. J. Med. Chem. 45, 2615–2623. 10.1021/jm020017n 12036371

[B129] Velliangiri S. Alagumuthukrishnan S. &Thankumar joseph S. I. (2019). A review of dimensionality reduction techniques for efficient computation. Procedia Comput. Sci., 165, 104–111. 10.1016/j.procs.2020.01.079

[B130] Viganò E. L. Ballabio D. &Roncaglioni A. (2024). Artificial intelligence and machine learning methods to evaluate cardiotoxicity following the adverse outcome pathway frameworks. Toxics 12 (1), 87. 10.3390/toxics12010087 38276722 PMC10820364

[B131] Wahaab A. Mustafa B. E. Hameed M. Stevenson N. J. Anwar M. N. Liu K. (2022). Potential role of Flavivirus ns2b-ns3 proteases in viral pathogenesis and anti-Flavivirus drug discovery employing animal cells and models: a review. Viruses 14 (1), 44–27. 10.3390/v14010044 PMC8781031 35062249

[B132] Wang C. Greene D. Xiao L. Qi R. Luo R. (2018a). Recent developments and applications of the MMPBSA method. Front. Mol. Biosci. 4 (JAN), 87. 10.3389/fmolb.2017.00087 29367919 PMC5768160

[B133] Wang G. Y. Zheng H. H. Zhang K. Y. Yang F. Kong T. Zhou B. (2018b). The roles of cytochrome P450 and P-glycoprotein in the pharmacokinetics of florfenicol in chickens. Iran. J. Veterinary Res. 19 (1), 9–14. 10.22099/ijvr.2018.4761 PMC5960766 29805456

[B134] Wesonga R. M. Awe O. I. (2022). An assessment of traditional and genomic screening in newborns and their applicability for Africa. Inf. Med. Unlocked 32, 101050. 10.1016/j.imu.2022.101050

[B135] Whitehorn J. Nguyen C. V. V. Khanh L. P. Kien D. T. H. Quyen N. T. H. Tran N. T. T. (2016). Lovastatin for the treatment of adult patients with dengue: a randomized, double-blind, placebo-controlled trial. Clin. Infect. Dis. Official Publ. Infect. Dis. Soc. Am. 62 (4), 468–476. 10.1093/cid/civ949 PMC4725386 26565005

[B136] Wlodawer A. Minor W. Dauter Z. &Jaskolski M. (2008). Protein crystallography for non-crystallographers, or how to get the best (but not more) from published macromolecular structures. FEBS J. 275 (1), 1–21. 10.1111/j.1742-4658.2007.06178.x PMC4465431 18034855

[B137] Wu W. &Herath A. (2016). “Chemometrics and predictive modelling,” in Nonclinical statistics for pharmaceutical and biotechnology industries. Editor Zhang L. (Springer International Publishing), 653–673. 10.1007/978-3-319-23558-5_25

[B138] Wu W.-L. Ho L.-J. Chen P.-C. Tsai Y.-T. Hsu S.-T. Chang D.-M. (2011). Immunosuppressive effects and mechanisms of leflunomide in dengue virus infection of human dendritic cells. J. Clin. Immunol. 31 (6), 1065–1078. 10.1007/s10875-011-9578-7 21845515

[B139] Xu T.-L. Han Y. Liu W. Pang X.-Y. Zheng B. Zhang Y. (2018). Antivirus effectiveness of ivermectin on dengue virus type 2 in *Aedes albopictus* . PLoS Neglected Trop. Dis. 12 (11), e0006934. 10.1371/journal.pntd.0006934 PMC6277121 30452439

[B140] Yadouleton A. Nouatin O. Kissira I. Houngbegnon P. Cottrell G. Fievet N. (2024). Genomic surveillance of dengue virus in Benin. Infect. Genet. Evol. J. Mol. Epidemiol. Evol. Genet. Infect. Dis. 125, 105674. 10.1016/j.meegid.2024.105674 39342977

[B141] Yap C. W. (2011). PaDEL-descriptor: an open source software to calculate molecular descriptors and fingerprints. J. Comput. Chem. 32 (7), 1466–1474. 10.1002/jcc.21707 21425294

[B142] Yuan S. Chan H. C. S. Hu Z. (2017). Using PyMOL as a platform for computational drug design. Wiley Interdiscip. Rev. Comput. Mol. Sci. 7 (2). 10.1002/wcms.1298

[B143] Zamri A. Teruna H. Y. Rahmawati E. N. Frimayanti N. &Ikhtiarudin I. (2019). Synthesis and *in silico* studies of a benzenesulfonyl curcumin analogue as a new anti dengue virus type 2 (DEN2) NS2B/NS3. Indonesian J. Pharm. 30 (2), 84–90. 10.14499/indonesianjpharm30iss2pp84-90

