# Supplementary material for: Machine learning and molecular docking prediction of potential inhibitors against dengue virus
Source: Front Chem. 2024 Dec 24;12:1510029. doi: 10.3389/fchem.2024.1510029 (PMC11703810; doi:10.3389/fchem.2024.1510029)
Supplement: Supplementary file 1 [file DataSheet1.docx]

**Supplementary Material**

**Machine Learning and Molecular Docking Prediction of Potential Inhibitors against Dengue Virus**

Supplementary table 1: The performance of the five predictive models based on the confusion matrix

| **Model** | **Accuracy** | **Precision** | **Recall** | **Specificity** | **F1 Score** | **False Positive Rate** | **False Negative Rate** | **True Positive Rate** | **True Negative Rate** |
| --- | --- | --- | --- | --- | --- | --- | --- | --- | --- |
| k-Nearest Neighbors (k-NN) | 0.916034 | 0.881133 | 0.699051 | 0.974563 | 0.779601 | 0.025437 | 0.300949 | 0.699051 | 0.974563 |
| Gaussian Naïve Bayes (NB) | 0.816941 | 0.596213 | 0.428481 | 0.921724 | 0.498619 | 0.078276 | 0.571519 | 0.428481 | 0.921724 |
| Support Vector Machine (SVM) | 0.928336 | 0.923544 | 0.722468 | 0.983867 | 0.810724 | 0.016133 | 0.277532 | 0.722468 | 0.983867 |
| Random Forest (RF) | 0.909042 | 0.923582 | 0.623418 | 0.986086 | 0.744379 | 0.013914 | 0.376582 | 0.623418 | 0.986086 |
| Logistic Regression (LR) | 0.952471 | 0.936632 | 0.832595 | 0.984806 | 0.881555 | 0.015194 | 0.167405 | 0.832595 | 0.984806 |

Supplementary table 2: The binding energies and intermolecular interactions between between the compounds and the NS2B/NS3 protease.

| **Compounds names** | **Binding Affinity (kcal/mol)** | **Hydrogen bonding with bond length (Å)** | **Hydrophobic Contacts** |
| --- | --- | --- | --- |
| anhydrophlegmacin | -9.2 | Asn152 (2.76), Gly153 (2.88), Ser135 (3.06), Gly151 (2.86) | Val72, Asp75, His51, Pro132, Tyr150, Leu128, |
| anhydrophlegmacin-9,10-quinones_B2 | -9.2 | Val72 (2.96), Asp75 (2.57), His51 (2.86), Lys73 (2.94) | Leu128, Pro132, Gly151, Gly153, Tyr161, Trp50 |
| ZINC000035941652 | -9.1 | Leu149 (3.06) | Trp83, Asn152, Ala164, Ile165, Lys73, Asn167, Thr120, Ile123, Ala166, Lys74, Gly148, Leu76 |
| chryslandicin | -9.0 | Val72 (2.74) | Gly153, Trp50, His51, Tyr161, Leu128, Pro132, Gly151, Asn152, Asp75 |
| ZINC000085594516 | -8.8 | Ser135 (3.09) | Leu128, Tyr150, Pro132, Phe130, Gly151, His51, Asn152, G1y153, Asp75 |
| 6a,12a-dehydromillettone | -8.7 | None | His51, Asp75, Gly151, Gly153, Tyr150, Phe130, Pro132, Leu128 |
| ZINC000028462577 | -8.6 | Ser135 (2.67), Val72 (2.94) | Trp50, Gly151, Leu128, Phe130, His51, Gly153, Pro132, Tyr150 |
| anhydrophlegmacin-9',10'-quinone | -8.6 | Asn152 (2.88), Gly153 (2.84), Ser135 (2.94) | Asp75, Val154, Val72, Trp50, His51, Pro132, Leu128, Gly151 |
| 2',4'-dihydroxychalcone-(4-O-5''')-4'',2''',4'''-trihydroxychalcone | -8.6 | Leu149 (2.99), Thr120 (3.26) | Val154, Lys73, Val72, Asn152, His51, Asp75, Gly148, Leu76, Gly153. Trp83, Lys74, Ile165, Ala166, Ala164, Asn167, Ile123 |
| ZINC000095485910 | -8.6 | Phe130 (2.71) | Ser135, Gly151, Leu128, His51, Asp75, Gly153, Pro132, Tyr150 |
| ZINC000095485955 | -8.6 | Trp83 (2.84), Leu149 (3.20), Asn152 (2.80) | Gly87, Val146, Met149, Leu76, Ala164, Asn167, Ile165, Ala166, Gly148, Leu85, Val147 |
| ZINC000095486025 | -8.5 | Leu128 (3.34) Gly153 (2.87) | Val72, His51, Asp75, Ser135, Gly151, Phe130, Pro132, Tyr150, Tyr161, Val54, Lys73, Asn152 |
| ZINC000038628344 | -8.5 | His51 (2.89), Ser135 (2.68), Asp75 (2.57), Phe130 (3.06), Tyr150 (3.10) | Pro132, Ser131, Leu128, Tyr161, Gly153, Gly151 |
| ZINC000095486053 | -8.4 | Gly151 (2.99) | His51, Pro132, Tyr150, Ser135, Phe130, Leu128 |
| phaseollidin | -8.4 | Gly87 (2.83), Val146 (2.98) | Leu85, Trp83, Gly148, Leu149, Ala164, Leu76, Asn167. Asn152, Lys74, Ile165, Trp89, Ala166, Glu88, Glu86, Val147 |
| 6-oxoisoiguesterin | -8.4 | Tyr150 (2.80, Phe130 (3.16, 2.83) | Ser131, Leu128, Gly151, Gly153, His51, Pro132 |
| ZINC000095486052 | -8.4 | Asn152 (3.20), Gly153 (3.14) | Pro132, Tyr150, Leu128, Tyr161, Gly151, His51, Asp75 |
| ZINC000014444870 | -8.4 | Asn152 (3.01), Leu149 (3.19) | Leu85, Val147, Gly87, Val146, Asn167, Ile165, Val54, Ala164, Ile123, Lys74, Gly148, Leu76, Trp83 |
| ZINC000095486076 | -8.4 | Leu149 (3.08), Asn152 (2.87), | Thr120, Lys73, Lys74, Asn167, Gly148, Leu76, Met49, Ile165, Ala164, Val154, Ile123 |
| liriodenine | -8.4 | Asn167 (3.11), Leu149 (3.11) | Lys74, Asn152, Leu76, Ile165, Ala164, Ala166, Leu85, Val147, Val146, Gly87, Trp83, Gly148 |
| 5,7'-physcion-fallacinol | -8.4 | Val72 (2.93), Ser135 (3.07), Phe130 (2.75), Tyr (3.24), Leu128 (2.91) | Asn152, His51, Pro132, Gly151, Gly153, Val154, Lys73 |
| ZINC000014677166 | -8.4 | Asn152 (3.10), Leu149 (2.86) Lys74 (3.19) | Gly148, Ile165, Val147, Asn167, Ala164, Val154, Lys73, Ile123, Met49, Leu76 |
| ZINC000014504006 | -8.3 | Lys74 (3.19), Asn152 (3.16), Leu149 (2.87) | Ala164, Met49, Val154, Ile123, Lys73, Asn167, Val147, Leu76, Trp83, Gly148 |
| ZINC000034165799 | -8.3 | Phe130 (3.10), Tyr150 (2.95) | Pro132, Ser131, Ser135, Gly151, His51, Gly153, Tyr161, Leu128 |
| corylin | -8.3 | Phe130 (2.86), Tyr150 (3.00) | Gly151, His51, Pro132, Leu128, Ser135, Asp75, Gly153 |
| lettowianthine | -8.3 | Gly153 (3.21) | Tyr161, Gly151, Ser135, Leu128, Tyr150, Pro132, Phe130 |
| ZINC000095485927 | -8.3 | Asn152 (2.70), Trp50 (3.08), Arg54 (2.96) | Asp75, Leu128, Ser135, Gly151, Pro132, Lys73, Gly153, His51 |
| ZINC000095485986 | -8.2 | Phe130 (2.85), Tyr150 (3.12), Val72 (2.79), Lys73 (2.96) | Lys74, Asp75, Tyr161, His51, Gly153, Ser131, Pro132, Leu128, Gly151, Ser135, |
| amentoflavone | -8.2 | Tyr150 (3.21), Gly153 (3.13), Ser135 (2.46) | Leu128, Pro132, Tyr161, Gly151, His51, Asp75, Asn152 |
| dihydrolanneaflavonol | -8.2 | Tyr150 (3.03), Phe130 (2.90), Gly151 (2.69), Ser135 (2.91), Asp75 (2.57, 3.00, 2.86), His51 (2.86, 2.57, 2.95) | Gly153, Leu128, Ser131, Pro132 |
| ZINC000095485907 | -8.2 | Val72 (3.03) | Asp75, His51, Tyr161, Leu128, Pro132, Tyr150, Gly151, Ser135, Gly153, Asn152 |
| ZINC000095485956 | -8.2 | Tyr150 (3.15), Gly153 (2.91) | Asp75, Asn152, Pro132, Ser135, Gly151, Leu128, Tyr161, His51 |
| ZINC000095486129 | -8.2 | Thr120 (3.12, 3.08), Lys73 (2.94, Lys73), Asn167 (2.80), Lys74 (2.81), Leu149 (3.23) | Gly148, Leu76, Ala166, Ile165, Ile123, Ala164, Val54, Asn152, Trp83, Val147 |
| voucapane-18,19-di-(4-methyl)-benzenesulphonate | -8.2 | None | Leu128, Tyr150, Pro132, Phe130, Tyr161, Gly153, Asp75, Trp50, His51, Gly151 |
| millettosine | -8.2 | None | Asp75, Gly151, Phe130, Tyr150, Leu128, Pro132, His51 |
| ZINC000095485958 | -8.1 | Ser135 (3.08), Tyr50 (2.88), Phe130 (2.51), Gly153 (3.19, 3.15) | Asn152, Asp75, Pro132, Tyr161, Ser131, Gly151, Leu128, His51 |
| chamuvaritin | -8.1 | Gly151 (2.98) | Tyr161, Gly153, His51, Val72, Asp75, Phe130, Tyr 150, Pro132, Leu128 |
| ZINC000014762512 | -8.1 | Leu149 (3.03, 2.76) | Gly87, Leu85, Leu76, Gly148, Met49, Ala164, Ala166, Asn152, Lys73, Lys74, Ile128, Val154, Asn167, Ile165, Val146, Typ83, Val147 |
| obovatachalcone | -8.1 | Asn152 (3.04), Ala166 (2.78), Asn167 (2.97) | Trp83, Lys74, Thr120, Lys73, Val154, Ile123, Ala164, Ile165, Leu76 |
| ZINC000095485940 | -8.1 | Ser135 (2.91) | Asp75, His51, Pro132, Tyr150, Phe130, Gly151, Leu128, Gly153, Tyr161 |
| ZINC000095486111 | -8.1 | Leu128 (2.86), Val72 (2.94) | Trp50, Asp75, His51, Gly151, Phe130, Pro132, Tyr150, Tyr161, Gly153 |
| epi-lupeol | -8.1 | Asn152 (3.21), Gly153 (2.78) | Tyr150, Pro132, Phe130, Leu128, His51, Asp75, Gly151, Tyr161 |
| ZINC000014779985 | -8.1 | Asn164 (2.89), Asn152 (2.80), Leu149 (3.04) | Leu76, Met49, Lys73, Val154, Ile123, Asn167, Lys74, Ala166, Ile165, Gly148, Trp83 |
| ZINC000095485990 | -8.1 | Leu149 (2.80), Asn167 (2.95) | Thr120, Ile123, Val154, Ile165, Asn152, Gly148, Ala164, Leu76, Lys74, Lys73 |
| ZINC000014825190 | -8.1 | Gly153 (2.91) | Arg54, Trp50, Val72, Asn152, Asp75, Leu128, Pro132, Gly151, His51 |
| ZINC000031168265 | -8.1 | Asp75 (3.08), Gly153 (3.24), Gly151 (3.03) | Tyr150, His51, Tyr161, Leu128, Pro132, Phe130 |
| ZINC000043069427 | -8.1 | Gly87 (3.05) | Val147, Leu149, Leu76, Asn152, Lys74, Lys73, Asn167, Thr120, Val154, Ile123, Ile165, Ala164, Ala166, Leu85, Trp83, Gly148 |
| (S)-lupinifolin_4'-methyl_ether | -8.0 | Tyr150 (2.90), Phe130 (3.18) | Leu128, Pro132, Gly151, His51, Ser135, Asp75, Gly153, Tyr161 |
| ZINC000004095704 | -8.0 | Gly153 (3.07, 2.90), Asp75 (2.94), Gly151 (3.28), Ser135 (2.94) | His51, Asn152, Phe130, Tyr150, Leu128 |
| 5beta,24-cyclofriedelan-3-one | -8.0 | Tyr150 (2.86) | His51, Pro132, Leu128, Phe130, Gly151, Gly153, Asp75 |
| ZINC000000006256 | -8.0 | Leu149 (3.04) | Gly87, Val147, Leu76, Val146, Trp83, Gly148, Ile165, Lys74, Ala164, Asn152 Asn167, Leu85 |
| chrysin | -8.0 | Leu149 (3.00, 2.80), Asn152 (2.81), Lys74 (3.28) | Gly148, Ala164, Ile123, Val154, Asn167, Ile165, Leu76 |
| ZINC000002026346 | -8.0 | None | Leu128, Tyr161, Gly151, Gly153, Asp75, Val72, His51, Pro132, Tyr150, Phe130 |
| ZINC000014780240 | -8.0 | Phe130 (2.83, 2.72), Tyr150 (2.93), Gly151 (2.80) | His51, Ser131, Pro132, Leu128, Tyr161, Gly153 |
| ZINC000014441502 | -8.0 | Gly151 (2.86), Ser135 (2.99) | Leu128, Gly153, Asn152, Val72, Asp75, His51, Phe130 |
| nordurlettone | -8.0 | Trp83 (2.92), Asn152 (3.28) | Gly148, Leu149, Ile165, Ala164, Val154, Ala166, Ile123, Thr120, Lys74, Asn167, Leu85, Leu76 |
| Leflunomide | -7.1 | None | Asn152, Val54, Ala64, Asn167, Leu76, Lys74, Ile123, Ala166 |
| Prednisolone | 7.0 | Gly151 (2.90, 2.71), Asp75 (2.95), His51 (3.21), Gly153 (2.93, 3.16) | Leu128, Phe130, Asn152, Ser135, Pro132 |

Supplementary table 3: ADME Prediction of selected hits and known inhibitors for Pharmacokinetics and physicochemical profiles.

| **Ligands** | **ESOL Solubility Class** | **GI absorption** | **RO5 violation** | **Veber’s rule violation** | **Mutagenicity** | **Tumorigenicity** |
| --- | --- | --- | --- | --- | --- | --- |
| ZINC000004095704 | Soluble | Low | 1 | 1 | None | None |
| ZINC000095485958 | Soluble | Low | 1 | 1 | None | None |
| ZINC000095485940 | Soluble | High | 0 | 0 | None | None |
| ZINC000095485986 | Soluble | Low | 0 | 1 | None | None |
| dihydrolanneaflavonol | Moderately soluble | High | 0 | 0 | None | None |
| lettowianthine | Moderately soluble | High | 0 | 0 | High | High |
| millettosine | Moderately soluble | High | 0 | 0 | None | None |
| ZINC000095486053 | Moderately soluble | High | 0 | 0 | None | None |
| ZINC000031168265 | Soluble | High | 0 | 0 | Noe | None |
| ZINC000095485910 | Moderately soluble | High | 0 | 0 | High | High |
| ZINC000014780240 | Moderately soluble | High | 0 | 0 | High | None |
| ZINC000085594516 | Poorly soluble | Low | 2 | 1 | None | None |
| 5,7'-physcion-fallacinol | Poorly soluble | Low | 2 | 1 | Low | None |
| ZINC000014441502 | Moderately soluble | High | 0 | 0 | None | None |
| chryslandicin | Poorly soluble | Low | 1 | 1 | None | High |
| 6a,12a-dehydromillettone | Moderately soluble | High | 0 | 0 | None | None |
| corylin | Moderately soluble | High | 0 | 0 | None | None |
| anhydrophlegmacin-9',10'-quinone | Poorly soluble | Low | 1 | 1 | Low | High |
| anhydrophlegmacin-9,10-quinones B2 | Poorly soluble | Low | 1 | 1 | Low | High |
| ZINC000038628344 | Moderately soluble | High | 0 | 0 | None | None |
| ZINC000095486025 | Moderately soluble | High | 0 | 0 | None | None |
| anhydrophlegmacin | Poorly soluble | Low | 1 | 1 | Low | High |
| ZINC000095485956 | Poorly soluble | Low | 2 | 1 | None | None |
| ZINC000014825190 | Moderately soluble | High | 0 | 0 | None | Low |
| ZINC000034165799 | Moderately soluble | High | 0 | 0 | None | None |
| ZINC000095486052 | Poorly soluble | High | 0 | 0 | None | None |
| amentoflavone | Poorly soluble | Low | 2 | 1 | None | Nonoe |
| (S)-lupinifolin 4'-methyl ether | Poorly soluble | High | 0 | 0 | None | None |
| ZINC000028462577 | Poorly soluble | Low | 1 | 1 | None | None |
| 2',4'-dihydroxychalcone-(4-O-5''')-4'',2''',4'''-trihydroxychalcone | Poorly soluble | Low | 1 | 1 | High | None |
| ZINC000095485907 | Moderately soluble | High | 1 | 0 | None | None |
| chamuvaritin | Poorly soluble | High | 0 | 0 | Low | Low |
| ZINC000002026346 | Poorly soluble | High | 0 | 0 | Low | Low |
| 6-oxoisoiguesterin | Poorly soluble | High | 1 | 0 | None | None |
| ZINC000095486111 | Poorly soluble | Low | 2 | 0 | None | None |
| epi-lupeol | Poorly soluble | Low | 1 | 0 | None | None |
| 5beta,24-cyclofriedelan-3-one | Poorly soluble | Low | 1 | 0 | None | None |
| voucapane-18,19-di-(4-methyl)-benzenesulphonate | Poorly soluble | Low | 2 | 0 | High | None |
| ZINC000095485927 | Insoluble | Low | 2 | 0 | None | None |

A)


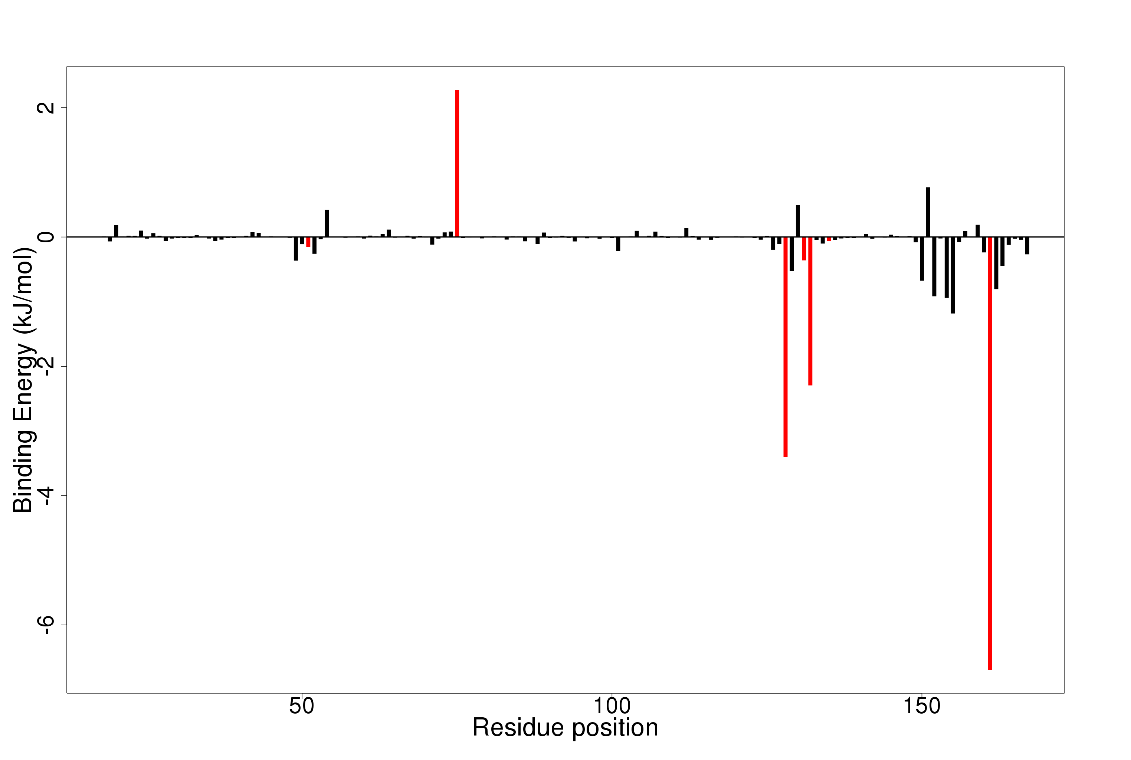


B)


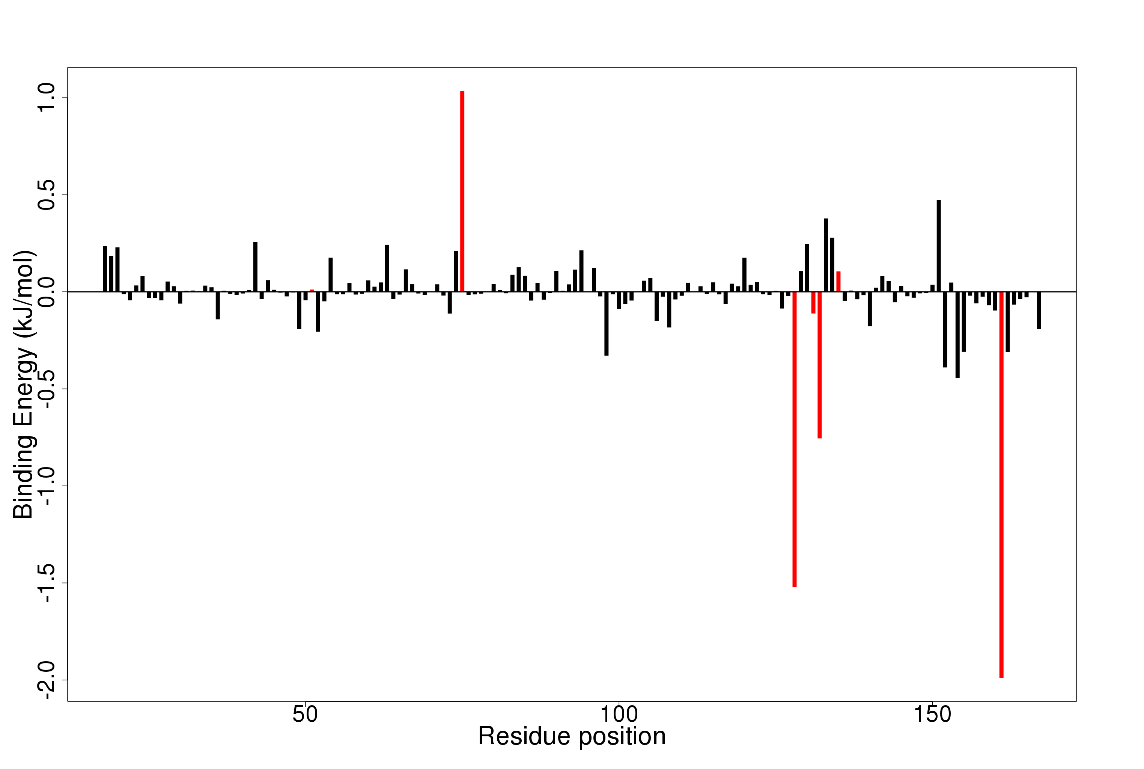


C)


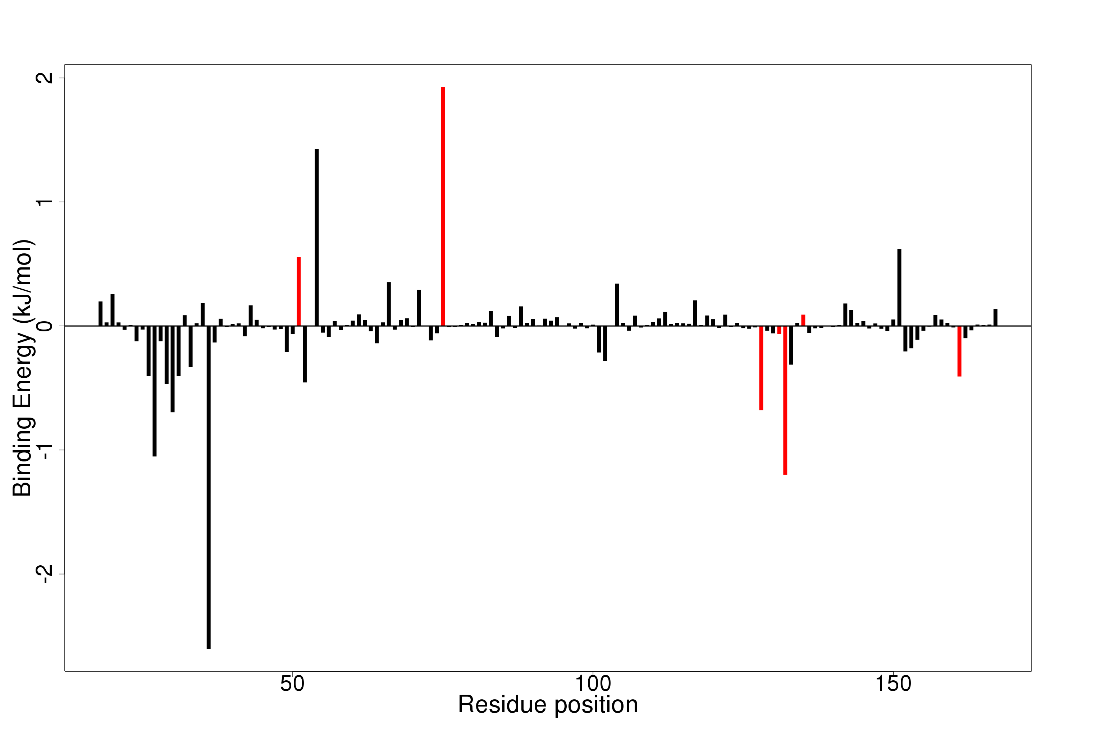


D)


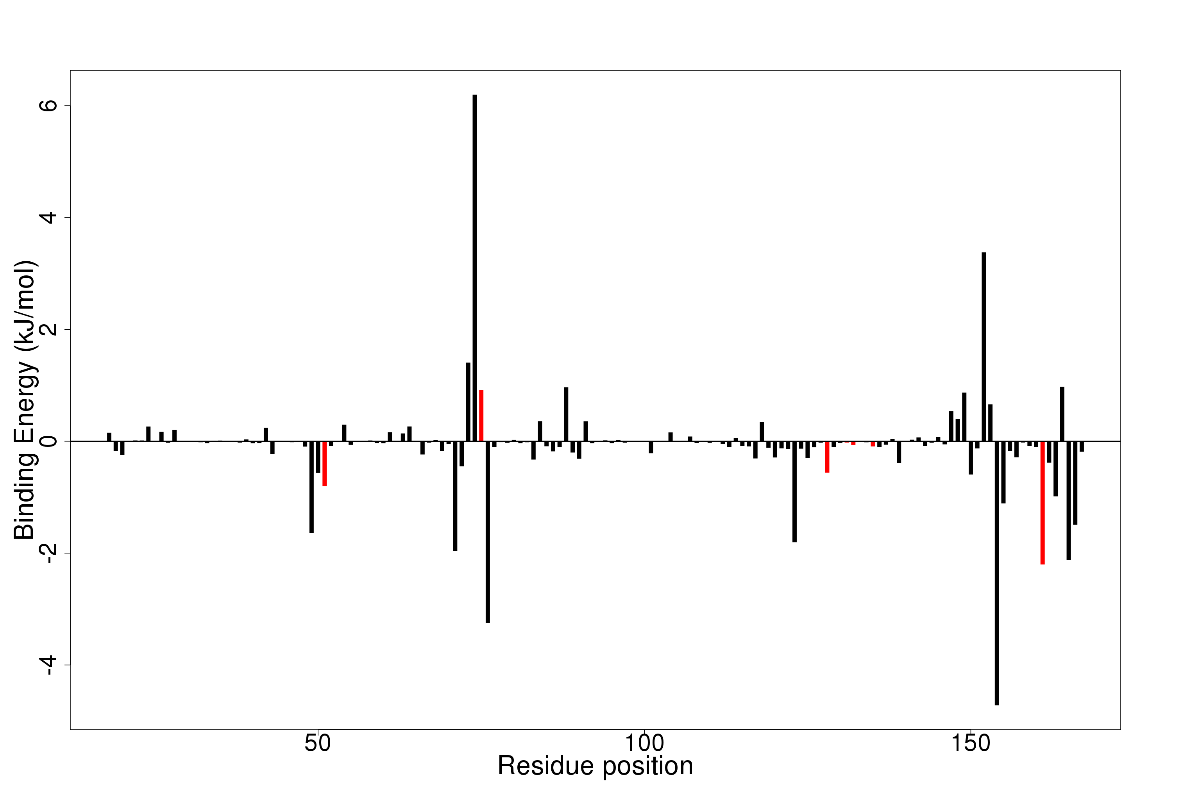


Supplementary Figure 1: Molecular mechanics Poisson-Boltzmann surface area (MM-PBSA) plot of binding free energy contribution per residue of protein-ligand complexes A) NS2B/NS3pro-ZINC38628344; B) NS2B/NS3pro-Prednisolone; C) NS2B/NS3pro-ZINC95485940; D) NS2B/NS3pro-2’,4’-dihydroxychalcone
